# Pathogenicity and Metabolites of *Purpureocillium lavendulum* YMF1.00683 against *Meloidogyne incognita*

**DOI:** 10.3390/pathogens11070795

**Published:** 2022-07-14

**Authors:** Zheng-Xue Bao, Rui Liu, Chun-Qiang Li, Xue-Rong Pan, Pei-Ji Zhao

**Affiliations:** State key Laboratory for Conservation and Utilization of Bio-Resources in Yunnan, School of Life Sciences, Yunnan University, Kunming 650091, China; bzx357489@126.com (Z.-X.B.); ruiliu991@outlook.com (R.L.); chunqianglee@outlook.com (C.-Q.L.); xuerong_pan@ynu.edu.cn (X.-R.P.)

**Keywords:** *Purpureocillium lavendulum*, pathogenicity, *Meloidogyne incognita*, avoidance effect, nematocidal activity

## Abstract

*Purpureocillium lavendulum* is a biological control agent with several registered products that can parasitize the eggs and larvae of various pathogenic nematodes. In this study, the pathogenicity and secondary metabolites of the fungus *P. lavendulum* YMF1.00683 were investigated. The strain YMF1.00683 had infection efficiency against the plant root-knot nematode *Meloidogyne incognita*. The strain’s process of infecting nematodes was observed under a microscope. Moreover, seven metabolites, including a new sterol (**1**), were isolated and identified from cultures of YMF1.0068 in Sabouraud’s dextrose agar. A bioassay showed that 5-methoxymethyl-1*H*-pyrrole-2-carboxaldehyde (**7**) is toxic to *M. incognita* and affects the egg hatching. It caused 98.23% mortality in *M. incognita* and could inhibit 80.78% of the hatching eggs at 400 μg/mL over a period of 96 h. Furthermore, 5-methoxymethyl-1*H*-pyrrole-2-carboxaldehyde (**7**) showed a strong avoidance effect at 40 ppm, and its chemotactic index value was −0.37. The results indicate that *P. lavendulum* could produce active metabolites against *M. incognita*.

## 1. Introduction

The damage caused by plant-parasitic nematodes to agriculture reaches USD157 billion every year, and the most serious damage is caused by the plant root-knot nematode belonging to the genus *Meloidogyne* [1,2]. Chemicals are still the most widely used pesticides in agriculture and forestry. However, long-term use of chemical nematicides may lead to the development of resistance among pathogens and may have detrimental effects on the environment and human health [3]; for example, methyl bromide is a broad-spectrum fumigant that is toxic to nematodes and has thus been banned. Currently, there is an urgent need for environmentally friendly biopesticide formulations, but their successful application is often affected by factors such as environmental conditions, storage time, and the degradation of the biocontrol strain. Therefore, one key measure to improve the effect and stability of biological pesticides is to analyze the control mechanism.

*Purpureocillium lavendulum* is a species of *Purpureocillium* (formerly *Paecilomyces*) that is taxonomically segregated from *Paecilomyces lilacinum* [4]. As a broad-spectrum biocontrol fungus (formerly *Paecilomyces*), it is effective against the parasitic nematodes [5,6,7], *Trialeurodes vaporariorum*, *Frankliniella occidentalis*, *Aphis gossypii*, *Tetranychus urticae* [8], and *Acromyrmex lundii* [9] and has good control effects, and it is widely available as a microbial pesticide on the market.

**Figure 1:**
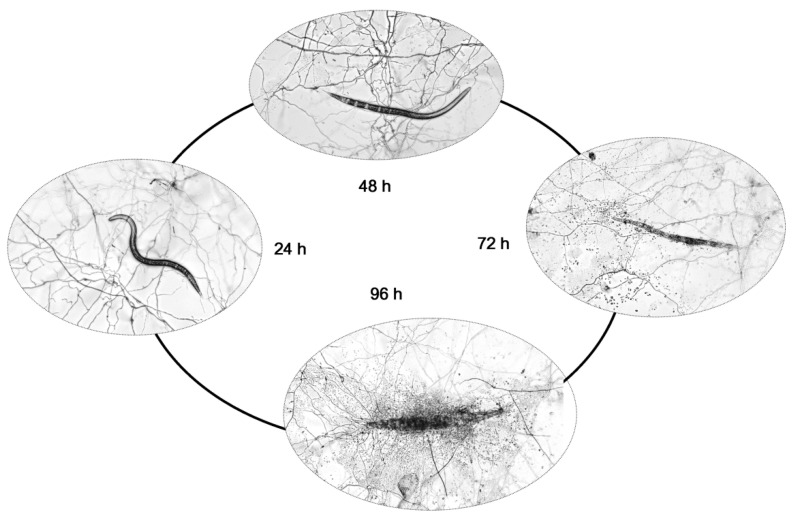
Infestation process of *M. incognita* by *P. lavendulum* YMF1.00683.

With the rapid development of whole-genome sequencing technology and informatics biology, the sequencing of numerous fungal genomes shows that most fungal metabolic biosynthetic potential has not been fully exploited. By analyzing the genomic data of *P. lavendulum* YMF1.00683 (which have not been released yet), it was found that this strain has the potential to produce abundant secondary metabolites. In general, the metabolites of microorganisms often play an important role in biological control of nematode. For example, 5-hydroxymethylfuran-2-carboxylic acid was isolated from *Drechmeria coniospora* and possessed nematocidal activity against *M. incognita* [10], and leucinostatins obtained from *Paecilomyces lilacinus* showed nematocidal activity [11], while other compounds such as phenolic acids and sesquiterpenoids were obtained from *Paecilomyces lilacinus* and exhibited nematocidal activity [11,12]. Therefore, it is necessary to carry out research on active metabolites from biocontrol potential fungus *P. lavendulum* YMF1.00683. In the present work, we observed the nematodes infected by *P. lavendulum* YMF1.00683 under a microscope and found that lethality gradually increased over time (Figure 1) during co-cultivation. We speculate that *P. lavendulum* YMF1.00683 produced small molecule compounds to kill the nematodes. Therefore, in this study, the metabolites were purified from culture extracts of *P. lavendulum* YMF1.00683, and we tested the compounds isolated for their activity against *M. incognita*. The results indicated that the metabolites from *P. lavendulum* YMF1.00683 showed multiple activities against nematodes.

## 2. Results

### 2.1. Pathogenicity of P. lavendulum YMF1.00683 against M. incognita

Many reports on fungi of the genus *Purpureocillium* (formerly *Paecilomyces*) have shown that they can control and antagonize root-knot nematodes of *M. incognita* [6,7]. Here we provide the microscopic observation of *P. lavendulum* YMF1.00683 infestations and the decomposition process of *M. incognita* in the laboratory. As shown in Figure 1, it can be seen that after three days of spore germination, the hyphae had overgrown the plate. At this time, approximately 50 *M. incognita* worms were added to each plate. After 24 h of *M. incognita* addition, the nematodes still had good viability. After 48 h, the vigor had been significantly reduced, and 20% of the *M. incognita* had died. At 72 h, 34% of *M. incognita* had died, and the body walls of the dead nematodes had ruptured. At 96 h, 41% of the nematodes had died, and the bodies of the dead nematodes had been degraded.

### 2.2. Isolation and Structural Identification of Compounds

**Figure 2:**
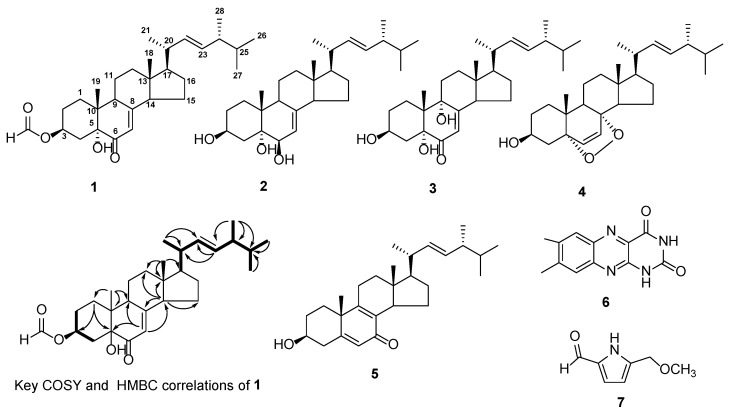
The structures of compounds **1**–**7** and key ^1^H−^1^H COSY (bold line) and HMBC (arrows) correlations of **1.** The figure includes five steroids (**1**–**5**), 7,8-dimethylalloxazine (**6**) and 5-methoxymethyl-1*H*-pyrrole-2-carboxaldehyde (**7**). In addition, the key ^1^H−^1^H COSY (**―**) and HMBC (**→**) correlations of **1** was shown in figure.

In order to investigate the metabolites and their function of *P. lavendulum* YMF1.00683, a systematic isolation of the extract of the strain with different chromatographic techniques is achieved. The extract (200.5 g) from the SD solid fermentation of YMF1.00683 was chromatographed on various columns (such as RP-18, Sephadex LH-20, and silica gel), and seven compounds (**1**–**7**) were obtained (Figure 2). Their structures were identified by NMR and MS data.

Compound **1** was obtained as a colorless solid, and its molecular formula was found to be C_29_H_44_O_4_ using high-resolution mass spectrometry 457.3311 ([M + H]^+^, Calcd. 457.3312); it contained eight unsaturation degrees. According to the nuclear magnetic resonance (NMR) spectrum data of compound **1** (Table 1), it contained 29 carbon signals, including 5 quaternary carbons, 11 methines, 7 methylenes, and 6 methyls.

NMR data analysis found that compound **1** was very similar to 3β,5α-dihydroxy-ergosta-7,22-dien-6-one [13], and the main difference was that the downfield shift of the 3-position in compound **1**. Compound **1**’s planar structure was determined from the key correlation points of the heteronuclear multiple bond correlation spectroscopy (HMBC) and data of 2D-NMR. In the COSY spectrum (Figure 2), the two branches were deduced to be –C-2−C-3−C-4− (−branch) and −C-17–C-20(-C-21)–C-22−C-23−C-24(C-28)–C-25(-C-27)–C-26−(−branch) from a complete interpretation of the key cross-peaks (H-2/H-3/H-4; H-17/H-20(/H-21)/H-22/H-23/H-24(/H-28)/H-25(/H-27)/ H-26). The HMBC experiment (Figure 2) showed that H-1 (δ_H_ 1.61 and 2.10) correlated with C-3 (δc 71.4) and C-5 (δc 77.0); H-4 (δ_H_ 2.16 and 2.74) correlated with C-3 (δc 71.4), C-5 (δc 77.0), and C-10 (δc 40.9); the olefinic proton H-7 (δ_H_ 5.90) was related to C-9 (δc 44.0), C-5 (δc 77.0), and C-14 (δc 55.6); H-9 (δ_H_ 2.92) correlated with C-18 (δc 16.0), C-11 (δc 21.9), C-10 (δc 40.9), C-7 (δc 120.2) and C-8 (δc 164.2); methyl protons H-19 (δ_H_ 1.02) correlated with C-1 (δc 30.5), C-10 (δc 40.9), C-9 (δc 44.0), and C-5 (δc 77.0); the methyl protons H-18 (δ_H_ 0.57) correlated with C-12 (δc 38.9), C-13 (δc 44.5), C-14 (δc 55.6), and C-17 (δc 55.9); the methyl protons H-21 (δ_H_ 1.03) correlated with C-20 (δc 40.5), C-17 (δc 55.9), and C-22 (δc 135.6); olefinic protons H-22 (δ_H_ 5.14) and H-23 (δ_H_ 5.26) correlated with C-20 (δc 40.5) and C-24 (δc 43.0); two methyls H-26 (δ_H_ 0.95) and H-27 (δ_H_ 0.85) correlated with C-25 (δc 33.2) and C-24 (δc 43.0). The formate proton H-29 (δ_H_ 8.31) correlated with C-3 (δc 71.4), which confirms that the formic acid group is connected with 3-OH by an ester bond (Figure 1). The NOESY experiment showed NOEs between H-3 and H-4α; H-4β and H-19. These data supported the relative configurations of C-3 and C-19 as well as the almost identical optical rotation values (3β,5α-dihydroxy-ergosta-7,22-dien-6-one, ${\left[\alpha \right]}_{D}^{19}$ = 26.5, c 0.1, CHCl_3_) indicated that the stereochemistry of **1** was the same as 3β,5α-dihydroxy-ergosta-7,22-dien-6-one [14]. Based on the similarity of the NMR data, optical rotation, and biogenetic considerations, **1** was proposed to have the absolute configuration shown in Figure 2.

The other metabolites were determined to be 3β,5α,6β-ergosta-7,22-dien-triol (**2**) [15], 3β,5α,9α-trihydroxy-ergosta-7,22-dien-6-one (**3**) [16], 5α,8α-epidioxy-22*E*-ergosta-6,22-dien-3β-ol (**4**) [17], 3β-hydroxy-(3,22*E*)-ergosta-5,8,22-trien-7-one (**5**) [18], 7,8-dimethylalloxazine (**6**) [19], and 5-methoxymethyl-1*H*-pyrrole-2-carboxaldehyde (**7**) [20] based on the reference data, and the NMR and MS spectra included in the Supplementary Materials.

### 2.3. Activity

#### 2.3.1. Effects of Compounds on the Egg Hatching of *M. incognita*

The hatching rate of eggs was the highest in the control group (1% MeOH water), and the hatching rates were 13.77%, 29.27%, 37.81%, and 47.39% at 24, 48, 72, and 96 h. The results of Tukey’s HSD test analysis by SPSS (IBM SPSS Statistics 26) (Table 2) showed that compounds **6** and **7** could significantly inhibit the egg hatching of *M. incognita* during all observation periods. However, other compounds did not affect egg hatching. With time extension, the hatching rate of nematode eggs increased.

#### 2.3.2. Evaluation of the Nematocidal Activity of Compounds against *M. incognita*

As shown in Table 3, at 400 ppm, the nematocidal activities of 5-(methoxymethyl)-1*H*-pyrrole-2-carboxaldehyde (**7**) were gradually enhanced with the increase in time, and compound **7** showed strong activity at 96 h (corrected mortality rate > 90%). We found, using multiple comparisons in SPSS (Table 3), that other compounds were not significantly different at the 0.05 level during all observation periods, and they showed moderate activity (30–40%) at 96 h. Meanwhile, the adjusted mortality of avermectin was 81% at a concentration of 100 μg/mL over a period of 48 h.

#### 2.3.3. Chemotaxis Activities of Compound **7** against *M. incognita*

**Figure 3:**
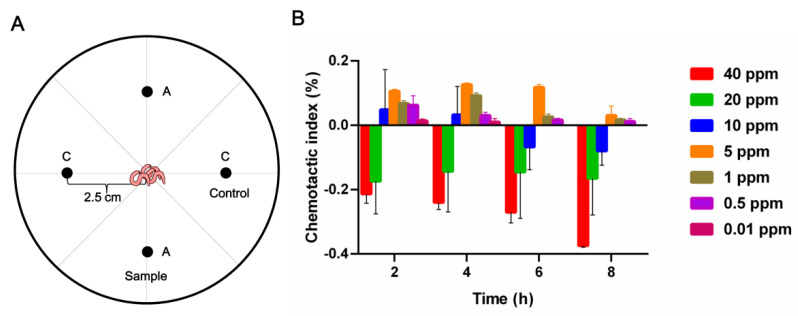
Effect of compound **7** on the chemotaxis of *M. incognita*. (**A**) Schematic representation of quadrant bioassay used to measure chemotaxis to compound **7**. The worms were placed in the plate center at the beginning of the assay. (**B**) Chemotaxis activity of **7** at different times and concentrations.

Furthermore, 5-(methoxymethyl)-1*H*-pyrrole-2-carboxaldehyde (**7**) showed obvious activity in inhibiting egg germination and nematocidal activity against *M. incognita* J2. Therefore, it was selected for the analysis of its chemotactic effect on *M. incognita*. The results showed that when the compound’s concentration was 40 ppm (Figure 3A), it exhibited a strong avoidance effect towards root-knot nematodes of *M. incognita* (CI < 0). When the compound’s concentration decreased to 5 ppm (Figure 3B), it showed a weaker attractive effect (0 < CI < 0.2).

## 3. Discussion

Root-knot disease caused by *M. incognita* is a serious concern because it affects several economically important crops globally. Researchers found that *Paecilomyces lilacinus* (currently valid name: *Purpureocillium lilacinum*) showed a parasitic effect on root-knot nematodes [6,7,8]. Several experts have conducted numerous studies on the fungus and have confirmed that *Paecilomyces lilacinus* has control effects on various plant-parasitic nematodes [3,21]. *Paecilomyces lilacinus* can parasitize the eggs and larvae of important pathogenic nematodes, including root-knot nematodes and cyst nematodes. *Paecilomyces lilacinus* was subsequently found to disrupt the lipid and chitin layers of nematode eggshells [22]. Additionally, *Paecilomyces lilacinus* can also produce a variety of biologically active secondary metabolites, including polyketides and non-ribosomally synthesized peptides, such as leucinostatins, which are nematocidal, antiviral, and phytotoxic and possess a series of biological activities [11,23,24]. By comparing the 18S rRNA gene, internal transcribed spacer, and partial translation elongation factor 1-a of the isolates of *P. lilacinus* from the environment and organisms, the genus *Purpureocillium* was proposed [4], which includes *P. lilacinum*, *P. lavendulum*, and *P. takamizusanense* [4,25].

While studying the infection of *M. incognita* by *P. lavendulum*, a certain percentage of nematodes died gradually over time (Figure 1) after the co-cultivation of *Pur. lavendulum* with *M. incognita*. Through the isolation, purification, and activity assaying of the compounds from the solid fermentation medium, two compounds showed activity against *M. incognita*. Among them, 7,8-dimethylalloxazine (6) exhibited weak egg hatching inhibitory activity; this is the main photodegradation product of riboflavin under neutral or acidic conditions and is known as an effective photosensitizer [26,27,28]. Some microorganisms and plants also produce 7,8-dimethylalloxazine [29,30] and show various types of activity: 7,8-dimethylalloxazine was isolated from culture filtrates of *Chlamydomonas* as a quorum-sensing signal-mimicking compound capable of activating *Pseudomonas aeruginosa* LasR receptor [31], and it was also purified and identified from a culture of the bacterium *Sinorhizobium meliloti* and found to be responsible for stimulating root respiration and plant growth. Moreover, 5-methoxymethyl-1*H*-pyrrole-2-carboxaldehyde (7), which was isolated from plants and other fungi [32,33], showed multiple activities in the present work: at a low concentration of 5 ppm, the metabolite showed a certain attraction to *M. incognita*, while at 40 ppm, it had a strong avoidance effect. Lastly, this compound showed nematocidal activity and inhibited egg hatching activity in *M. incognita* at a concentration of 400 ppm.

## 4. Materials and Methods

### 4.1. Materials

Optical rotations were measured with a Jasco DIP-370 digital polarimeter (JASCO, Tokyo, Japan). Ultraviolet (UV) spectra were recorded on a Shimadzu UV-2401PC spectrophotometer. The nuclear magnetic resonance (NMR) spectra were recorded on Avance III-600 spectrometers, with tetramethylsilane (TMS) as an internal standard. The electrospray ionization mass spectra (ESI-MS) and high-resolution electrospray ionization mass spectra (HR-ESI-MS) were recorded on a high-resolution Thermo Q Exactive Focus mass spectrometer (Thermo Fisher Scientific, Bremen, Germany).

Column chromatography was performed on silica gel G (200–300 mesh), GF254 (Qingdao Marine Chemical Inc., Qingdao, China), and Sephadex LH-20 (Amersham Pharmacia, Chicago, IL, USA). Precoated silica gel GF254 plates (Qingdao Marine Chemical Inc., Qingdao, China) were used for thin-layer chromatography (TLC). Fractions were monitored by TLC and visualized using heating plates sprayed with 5% H_2_SO_4_ in EtOH.

*P. lavendulum* YMF1.00683 was isolated from a soil sample in Yunnan. After 8 days of growth on PDA at 28 °C, the hyphae of *P. lavendulum* YMF1.00683 have covered 6 cm plates, and it is no growth at 35 °C. Colonies consist of dense basal mats composed of many conidiophores and sparse aerial hyphae, and they fold radially toward the periphery. Conidiophores grow from the mycelium 3–4 days at 28 °C, are long stem chains, unicellular, subglobose, apically or lemon-colored at the base, and the clumps are pinkish-purple [34]. *P. lavendulum* YMF1.00683 was stored in glycerol at −80 °C at the State Key Laboratory for Conservation and Utilization of Bio-Resources in Yunnan, China. The YMF1.00683 strain was inoculated in PDA medium and incubated at 28 °C for 6 days, and then was transferred into a fresh medium for 5 days as a seed strain. *Meloidogyne incognita* was obtained from the roots of tomatoes grown in E’shan County in Yunnan Province.

### 4.2. Infection of M. incognita by P. lavendulum

*P. lavendulum* grown on PDA plates for seven days, was harvested by cutting the sample into pieces, shaking it at 100 RPM in 0.1% Tween−80 for 30 min, and filtering it through sterilized four-layer filter paper to obtain a spore suspension. The spores were counted, and their concentration was adjusted to 10^6^ mL^−1^. A 200 μL aliquot of the spore suspension was dispersed on a cellophane-covered water agar plate, which was then cultured at 28 °C for three days to germinate spores. *M. incognita* was washed with sterile ddH_2_O, and then approximately 50 *M. incognita* worms were added to the germinated spore plates. The infection process and number of dead nematodes were observed and recorded at 24, 48, 72, and 96 h.

### 4.3. Extraction and Separation of Metabolites

The mycelium of *Pur. lavendulum* was cultivated in SD solid medium (10 g tryptone, 40 g glucose, 15 g agar, 1 L water) for 21 days at 28 °C. The culture (30 L) was cut into small pieces and extracted by EtOAc/MeOH/AcOH (80:15:5, *v*/*v*/*v*) 3 times, and then concentrated under reduced pressure to obtain a crude extract. The crude extract was dissolved with 2.5 L of pure water, extracted with EtOAc to obtain the EtOAc crude extract (100.2 g), and then extracted with *n*-butanol to obtain the *n*-butanol extract (100.3 g). After LC-MS analysis, both extracts were combined.

The whole crude extract (200.5 g) was placed on a reversed-phase silica gel column (300 g) and eluted with H_2_O/MeOH mixtures (100:0, 90:10, 70:30, 50:50, 30:70, 10:90, and 0:100) to obtain 10 fractions Fr.1–Fr.10. Fr.10 (3.28 g) was separated by Sephadex LH-20 (chloroform–methanol, 1:1) to obtain 4 fractions (Fr.10.1–Fr.10.4). Fr.10.3 (534 mg) was chromatographed on a silica gel column (200–300 mesh) and eluted with a gradient of petroleum ether–acetone (500:1 to 7:3 with 0.1% NH_3_·H_2_O) to give Fr.10.3.1–Fr.10.3.10. Fr.10.3.8 (37 mg) was separated by a silica gel column (200–300 mesh) with petroleum ether–ethyl acetate (400:1 to 7:3) and then purified with Sephadex LH-20 (methanol) to give **4** (3 mg). Fr.8 (1.25 g) was subjected to Sephadex LH-20 (chloroform–methanol, 1:1) to give 5 fractions (Fr.8.1–Fr.8.5). Fr.8.3 (609 mg) was separated using Sephadex LH-20 (chloroform–methanol, 1:1) to give Fr.8.3.1–Fr.8.3.8. Fr.8.3.1 (37 mg) was separated by a silica gel column (200–300 mesh) and eluted by a petroleum ether–acetone (50:1 to 6:4), and then purified by a silica gel column (200–300 mesh, chloroform–methanol, 20:1 to 8:2) to give compound **1** (4 mg). Fr.8.3.4 (6 mg) was chromatographed on a silica gel column (200–300 mesh) eluting with chloroform–acetone (200:1 to 6:4) and then purified by Sephadex LH-20 (acetone) to obtain compound **3** (2 mg). Fr.8.3.5 (9 mg) was placed on a silica gel column (200–300 mesh) and eluted with petroleum ether-acetone (500:1 to 1:1) to give compound **2** (5 mg). Fr.8.3.8 (43 mg) was placed on a silica gel column (200–300 mesh) and eluted with chloroform–acetone (100:1 to 8:2), and then purified by Sephadex LH-20 (methanol) to obtain compound **5** (2 mg). Fr.4 (4.27 g) was chromatographed on Sephadex LH-20 (chloroform–methanol, 1:1) to give 4 fractions (Fr.4.1–Fr.4.4). Fr.4.4 (10 mg) was separated on a silica gel column (200–300 mesh) and eluted with chloroform-acetone (200:1 to 6:4), and then purified by Sephadex LH-20 (methanol) to obtain compound **6** (8 mg). Fr.3 (3.76 g) was subjected to Sephadex LH-20 (chloroform–methanol, 1:1) to give 5 fractions (Fr.3.1–Fr.3.5). Fr.3.4 (162 mg) was separated on a silica gel column (200–300 mesh) and eluted with chloroform–methanol (50:1 to 10:1), and then purified by Sephadex LH-20 (methanol) to obtain compound **7** (5 mg).

### 4.4. Spectroscopic Data

Compound **1**: colorless solid; ${\left[\alpha \right]}_{D}^{19}$ = 25.8 (*c* 0.10, MeOH); UV (MeOH) *λ*_max_ (log ε) nm: 201 (3.86), 251 (3.92); NMR data see Table 1. ESI-MS *m*/*z*: 457 [M + H]^+^; HR-ESI-MS: 457.3311 ([M + H]^+^, calc. for C_29_H_44_O_4_, 457.3312).

3β,5α,6β-ergosta-7,22-dien-triol (**2**), colorless solid; C_28_H_46_O_3_; ESI-MS *m*/*z*: 443 [M + Na]^+^. ^1^H-NMR (C_5_D_5_N, 600 MHz) δ: 4.85 (1H, m, H-3), 4.32 (1H, m, H-6), 5.74 (1H, brd, *J* = 2.9 Hz, H-7), 5.14 (1H, dd, *J* = 8.4, 15.2 Hz, H-22), 5.21 (1H, dd, *J* = 7.6, 15.2 Hz, H-23); ^13^C-NMR (C_5_D_5_N, 150 MHz) δ: 32.5 (C-1), 33.7 (C-2), 67.4 (C-3), 41.9 (C-4), 76.0 (C-5), 74.1 (C-6), 120.4 (C-7), 141.4 (C-8), 42.9 (C-9), 37.9 (C-10), 22.3 (C-11), 39.7 (C-12), 43.5 (C-13), 55.1 (C-14), 23.3 (C-15), 28.4 (C-16), 55.9 (C-17), 12.4 (C-18), 18.7 (C-19), 40.8 (C-20), 20.3 (C-21), 136.2 (C-22), 131.9 (C-23), 43.1 (C-24), 33.4 (C-25), 20.0 (C-26), 19.7 (C-27), 20.0 (C-28).

3β,5α,9α-trihydroxy-ergosta-7,22-dien-6-one (**3**): colorless solid; ESI-MS *m*/*z*: 445 [M + H]^+^. ^1^H-NMR (CDCl_3_, 600 MHz) δ: 4.06 (2H, m, H-3), 5.67 (1H, s, H-7), 1.03 (3H, s, H-18), 0.62 (3H, s, H-19), 1.02 (3H, d, *J* = 6.9 Hz, H-21), 5.25 (2H, m, H-23/H-24), 0.83 (3H, d, *J* = 6.9 Hz, H-26), 0.84 (3H, d, *J* = 6.9 Hz, H-27), 0.92 (3H, d, *J* = 6.9 Hz, H-28); ^13^C-NMR (CDCl_3_, 150 MHz) δ: 25.4 (t, C-1), 29.6 (t, C-2), 67.2 (d, C-3), 33.0 (t, C-4), 79.7 (s, C-5), 197.6 (d, C-6), 119.9 (d, C-7), 164.3 (s, C-8), 74.7 (d, C-9), 41.8 (s, C-10), 28.8 (t, C-11), 34.9 (t, C-12), 45.3 (s, C-13), 51.7 (d, C-14), 22.4 (t, C-15), 27.9 (t, C-16), 56.0 (d, C-17), 21.1 (q, C-18), 12.2 (q, C-19), 40.3 (d, C-20), 19.6 (q, C-21), 135.0 (d, C-22), 132.4 (d, C-23), 42.8 (d, C-24), 33.0 (d, C-25), 19.9 (q, C-26), 20.4 (q, C-27), 17.6 (q, C-28).

5α,8α-epidioxy-22E-ergosta-6,22-dien-3β-ol (**4**): colorless solid; ESI-MS *m*/*z*: 451 [M + Na]^+^. ^1^H-NMR (CDCl_3_, 600 MHz) δ: 3.95 (1H, m, H-3), 6.25 (1H, d, *J* = 8.4 Hz, H-6), 6.50 (1H, d, *J* = 8.5 Hz, H-7), 5.21 (1H, dd, *J* = 8.6, 15.3 Hz, H-23), 5.16 (1H, dd, *J* = 8.6, 15.3 Hz, H-22), 1.00 (3H, d, *J* = 6.6, H-21), 0.91 (3H, d, *J* = 6.7, H-28), 0.88 (3H, s, H-19), 0.84 (3H, d, J = 6.7 Hz, H-27), 0.82 (3H, d, *J* = 6.8 Hz, H-18), 0.81 (3H, s, H-28); ^13^C-NMR (CDCl_3_, 150 MHz) δ: 34.7 (t, C-1), 30.1 (t, C-2), 66.5 (d, C-3), 36.9 (t, C-4), 82.1 (s, C-5), 135.4 (d, C-6), 130.7 (d, C-7), 79.4 (s, C-8), 51.7 (d, C-9), 36.9 (s, C-10), 23.4 (t, C-11), 39.7 (t, C-12), 44.5 (s, C-13), 51.7 (d, C-14), 20.8 (t, C-15), 28.7 (t, C-16), 56.2 (d, C-17), 12.9 (q, C-18), 18.2 (q, C-19), 39.7 (d, C-20), 20.9 (q, C-21), 135.2 (d, C-22), 132.3 (d, C-23), 42.8 (d, C-24), 33.0 (d, C-25), 19.9 (q, C-26), 19.6 (q, C-27), 17.5 (q, C-28).

3β-hydroxy-(3,22*E*)-ergosta-5,8,22-trien-7-one (**5**): colorless solid; C_28_H_42_O_2_; ESI-MS *m*/*z*: 411 [M + H]^+^. ^1^H-NMR (CDCl_3_, 600 MHz) δ: 6.04 (1H, s, H-6), 5.22 (2H, m, H-22/H-23), 3.67 (1H, m, H-3), 1.28 (3H, s, H-19), 0.99 (3H, d, *J* = 6.6 Hz, H-21), 0.86 (3H, d, *J* = 6.8 Hz, H-28), 0.78 (3H, d, *J* = 6.8 Hz, H-27), 0.76 (3H, d, *J* = 6.8 Hz, H-26), 0.58 (3H, s, H-18); ^13^C-NMR (CDCl_3_, 150 MHz) δ: 186.3 (s, C-7), 71.9 (d, C-3), 161.5 (s, C-5), 161.0 (s, C-9), 126.8 (d, C-6), 135.4 (d, C-8), 24.6 (t, C-1), 29.5 (t, C-2), 33.1 (t, C-4), 41.8 (s, C-10), 24.6 (t, C-11), 34.6 (t, C-12), 42.3 (s, C-13), 48.4 (d, C-14), 22.4 (t, C-15), 27.9 (t, C-16), 53.3 (d, C-17), 21.1 (q, C-18), 11.9 (q, C-19), 40.3 (d, C-20), 19.6 (q, C-21), 134.0 (d, C-22), 132.1 (d, C-23), 42.8 (d, C-24), 33.0 (d, C-25), 19.6 (q, C-26), 20.0 (q, C-27), 17.6 (q, C-28).

7,8-dimethylalloxazine (**6**): yellow crystal; ESI-MS *m*/*z*: 243 [M + H]^+^. ^1^H-NMR (C_5_D_5_N, 600 MHz) δ: 8.03 (1H, s, H-9), 7.85 (1H, s, H-6), 2.30 (3H, s, 8-Me), 2.23 (s, 7-Me); ^13^C-NMR (C_5_D_5_N, 150 MHz) δ: 162.0 (s, C-3), 151.7 (s, C-2), 147.6 (s, C-9a), 144.7 (s, C-7), 142.9 (s, C-10a), 139.7 (s, C-8), 139.0 (s, C-5a), 130.9 (s, C-4a), 127.1 (d, C-9), 123.7 (d, C-6), 20.3 (q, C-11), 19.7 (q, C-12).

5-(methoxymethyl)-1*H*-pyrrole-2-carboxaldehyde (**7**): pale-yellow oil; ESI-MS *m*/*z*: 140 [M + H]^+^. ^1^H-NMR (CD_3_OD, 600 MHz) δ: 3.34 (3H, s, OCH_3_), 4.44 (2H, s, H-6), 6.27 (1H, d, *J* = 3.8 Hz, H-4), 6.96 (1H, d, *J* = 3.8 Hz, H-3), 9.39 (1H, s, H-1); ^13^C-NMR (CD_3_OD, 150 MHz) δ: 180.7 (d, C-1), 139.7 (s, C-5), 134.4 (s, C-2), 121.5 (d, C-3), 111.8 (d, C-4), 67.7 (t, C-6), 58.3 (q, OCH_3_).

### 4.5. Assay Activity against M. incognita

#### 4.5.1. Effects of Compounds on the Hatching of *M. incognita* Eggs

Assays were performed in 24-well cell culture plates. *M. incognita* egg acquisition [10]: oocysts were picked from the root knots of susceptible tomato and washed of impurities with ddH_2_O, shaken with 0.5% NaClO for 3 min, and then filtered with a 500-mesh sieve to remove NaClO, rinsed with sterile water 3 times, shaken with 2% NaClO solution for 3 min to obtain eggs, and subjected to 10 μL microscopic examinations after completion to prevent excessive cracking. For the second stage juveniles of *M. incognita* (J2), the obtained eggs were hatched in a 25 °C incubator, and *M. incognita* (J2) were collected for later use.

The experimental operation of the effect on the egg hatching of *M. incognita* [35]: we used sterile water to prepare the egg solution; we prepared a compound solution with methanol sterile water, and the final concentration of methanol was 1%; egg-containing solution and compound solution were added to each well for a final total volume of 500 μL, containing approximately 150 eggs, and the tested compound working concentration was 400 ppm. Sterile water containing 1% methanol was used as a control, and each treatment was repeated thrice.

#### 4.5.2. Assay of Nematocidal Activity against *M. incognita*

The tested compounds were dispersed in MeOH. Two hundred *M. incognita* J2s (100 μL) were added to each sample, and the final concentration of the tested compounds was set at 400 ppm. The total and dead nematode numbers were determined every 24 h [36]. The worms were considered dead if they were flat or cracked, and then the nematode mortality was calculated. According to the nematocidal activity strength classification standard, the five-level grading criteria are as follows: inactive, adjusted mortality rate ≤10%; weak activity, adjusted mortality rate of 10% to 30%; moderate activity, adjusted mortality rate of 30% to 70%; strong activity, adjusted mortality > 70%. Avermectin was used as a positive control; the test solution without compound was used as a negative control. Three replicates were conducted for each test.

#### 4.5.3. Assay of Chemotaxis Activity against *M. incognita*

The nematode chemotaxis assay was conducted for compound **7** using the four-point plate method [37]. The 90 mm plate contained 1% agarose for the chemotaxis assay. Compound **7**’s working concentration was set to 40, 20, 10, 5, 1, 0.5, and 0.01 ppm using 1% methanol sterile water, and sterile water containing 1% methanol was used as a control. Approximately 200 worms of *M. incognita* J2 were added to the center of the Petri dish. Compound **7** and control were added, as shown in the schematic diagram (Figure 3A), the counts were observed at 2, 4, 6, and 8 h, and the chemotaxis index (CI) was calculated.

## Figures and Tables

**Table 1:** The NMR data of compound **1** (C_5_D_5_N, 600 MHz).

Position	^1^H	^13^C	HMBC
1	1.61 (1H, m)	30.5, t	C-3, C-5
	2.10 (1H, dt, J = 3.8, 9.7 Hz)		C-2, C-3, C-5, C-10
2	1.61 (1H, m)	27.1, t	-
	2.00 (1H, m)		-
3	5.76 (1H, m)	71.4, d	-
4	2.16 (1H, dd, J = 11.8, 13.1 Hz)	33.5, t	C-3, C-5, C-10
	2.74 (1H, dd, J = 5.0, 13.1 Hz)		C-3, C-5, C-10
5	-	77.0, s	-
6	-	199.0, s	-
7	5.90 (1H, s)	120.2, d	C-5, C-9, C-14
8	-	164.2, s	-
9	2.92 (1H, m)	44.0, d	C-19, C-11, C-10, C-8, C-7
10	-	40.9, s	-
11	1.46 (2H, m)	21.9, t	-
	2.00 (1H, m)		-
12	1.56 (1H, m)	38.9, t	-
	2.00 (1H, m)		-
13	-	44.5, s	-
14	1.98 (1H, m)	55.6, d	C-7, C-8, C-18, C-15, C-12, C-13
15	1.45 (2H, m)	22.6, t	C-13, C-14
16	1.68 (1H, m)	28.1, t	C-18, C-13, C-14, C-17
	2.10 (1H, m)		C-18, C-13, C-14
17	1.21 (1H, m)	55.9, d	C-16, C-13
18	0.57 (3H, s)	12.6, q	C-12, C-13, C-14, C-17
19	1.02 (3H, s)	16.0, q	C-1, C-10, C-9, C-5
20	2.00 (1H, m, overlap)	40.5, d	-
21	1.03 (3H, d, J = 6.6 Hz)	21.2, q	C-17, C-20, C-22
22	5.14 (1H, dd, J = 8.4, 15.2 Hz)	135.6, d	C-20, C-24, C-23
23	5.26 (1H, dd, J = 7.8, 15.2 Hz)	132.3, d	C-20, C-24, C-22
24	1.87 (1H, m, overlap)	43.0, d	-
25	1.45 (1H, m, overlap)	33.2, d	-
26	0.95 (3H, d, J = 6.5 Hz)	17.7, q	C-25, C-24, C-23
27	0.85 (3H, d, J = 6.6 Hz)	19.7, q	C-24, C-25, C-28
28	0.86 (3H, d, J = 6.7 Hz)	20.0, q	C-24, C-25, C-27
29	8.31 (1H, s)	161.2, d	C-3

**Table 2:** Effects of compounds on the egg hatching of *Meloidogyne incognita*.

Compounds		Hatching Rate % (Inhibition Rate %)		
	24 h	48 h	72 h	96 h
Control	13.77 ^b^	29.27 ^d^	37.81 ^d^	47.39 ^e^
**1**	12.79 ^b^ (9.8)	27.09 ^d^ (7.4)	37.28 ^d^ (1.40)	47.30 ^e^ (0.19)
**2**	11.98 ^b^ (13.0)	28.35 ^d^ (3.1)	36.95 ^d^ (2.27)	46.58 ^e^ (1.71)
**3**	11.92a ^b^ (13.44)	24.28 ^c^ (17.05)	37.21 ^d^ (1.59)	47.24 ^e^ (0.32)
**4**	12.36 ^b^(10.2)	29.05 ^d^ (0.75)	37.19 ^d^ (1.64)	47.21 ^e^(0.38)
**5**	10.38 ^ab^ (24.62)	22.15 ^b^ (24.33)	30.36 ^c^ (19.70)	39.40 ^d^ (16.86)
**6**	9.67 ^ab^ (29.77)	13.07 ^ab^ (55.59)	16.78 ^b^ (54.33)	20.79 ^c^ (56.13)
**7**	7.1 ^a^ (48.44)	12.36 ^a^ (57.77)	14.32 ^a^ (62.13)	19.22 ^b^ (59.44)

Note: In the same column of data, Tukey’s HSD test with different letters indicates significant differences (α = 0.05), and the numbers in brackets indicate the degree of inhibition of egg hatching compared with the control.

**Table 3:** Nematocidal activity of compounds against *M. incognita*.

Compounds	Adjusted Mortality (%)				
	12 h	24 h	48 h	72 h	96 h
Control	(2.06 ± 0.22) ^bc^	(2.07 ± 0.12) ^c^	(2.67 ± 0.58) ^d^	(4.40 ± 0.56) ^d^	(5.73 ± 1.10) ^e^
**1**	(0) ^c^	(2.50 ± 1.30) ^b^	(13.56 ± 1.90) ^b^	(22.83 ± 1.82) ^b^	(30.45 ± 2.05) ^c^
**2**	(0) ^c^	(2.38 ± 0.90) ^b^	(15.47 ± 1.90) ^b^	(21.76 ± 1.39) ^b^	(29.06 ± 1.98) ^c^
**3**	(1.87 ± 2.14) ^bc^	(7.03 ± 0.49) ^b^	(19.20 ± 2.21) ^b^	(27.70 ± 0.82) ^b^	(32.17 ± 2.32) ^c^
**4**	(0) ^c^	(2.63 ± 0.77) ^c^	(8.73 ± 1.08) ^d^	(17.60 ± 2.73) ^d^	(22.06 ± 2.4) ^d^
**5**	(0) ^c^	(2.83 ± 0.76) ^c^	(4.73 ± 1.00) ^d^	(14.60 ± 4.33) ^c^	(18.63 ± 2.01) ^d^
**6**	(2.90 ± 0.79) ^bc^	(3.67 ± 0.38) ^c^	(12.40 ± 2.19) ^c^	(16.47 ± 1.24) ^c^	(31.20 ± 1.82) ^c^
**7**	(23.20 ± 1.51) ^a^	(29.30 ± 2.33) ^a^	(41.60 ± 3.80) ^a^	(68.87 ± 3.63) ^a^	(98.23 ± 0.81) ^a^

Note: The data in the table are mean ± standard deviation. Different letters in the same column indicate significant differences at *p* < 0.05 by Duncan’s new multiple range test.

## Data Availability

All the data and methods necessary to reproduce this study are included in the manuscript and Supplemental Information.

## Supplementary Materials

The following supporting information can be downloaded at: https://www.mdpi.com/article/10.3390/pathogens11070795/s1. The HR-ESI-MS, 1D, and 2D NMR spectra of compound **1**, and ESI-MS and 1D NMR spectra of compounds **2**–**7**, are available in the Supporting Information.
