# Supplementary material for: Pathogenicity and Metabolites of Purpureocillium lavendulum YMF1.00683 against Meloidogyne incognita
Source: Pathogens. 2022 Jul 14;11(7):795. doi: 10.3390/pathogens11070795 (PMC9320282; doi:10.3390/pathogens11070795)
Supplement: Supplementary file 1 [file pathogens-11-00795-s001.zip › pathogens-1778221 Supplementary Materials.pdf]

Supplementary Materials for

Pathogenicity and Metabolites of *Purpureocillium lavendulum* YMF1.00683 against *Meloidogyne incognita*

Zheng-Xue Bao, Rui Liu, Chun-Qiang Li, Xue-Rong Pan, Pei-Ji Zhao*

State key Laboratory for Conservation and Utilization of Bio-Resources in Yunnan, School of Life Sciences, Yunnan University, Kunming, Yunnan 650091, China;

* Correspondence: P-J. Z. (email: pjzhao@ynu.edu.cn);

Contents of Supplementary Materials

Figure S1	^{1}H NMR spectrum of compound **1**	3
Figure S2	^{13}C NMR spectrum of compound **1**	4
Figure S3	HSQC spectrum of compound **1**	5
Figure S4.	HMBC spectrum of compound **1**	6
Figure S5	^{1}H–^{1}H COSY spectrum of compound **1**	7
Figure S6.	ROESY spectrum of compound **1**	8
Figure S7.	HR-ESI-MS of compound **1**	9
Figure S8	UV spectrum of compound **1**	10
Figure S9	^{1}H NMR spectrum of compound **2**	11
Figure S10	^{13}C NMR spectrum of compound **2**	12
Figure S11	ESI-MS spectrum of compound **2**	13
Figure S12	^{1}H NMR spectrum of compound **3**	14
Figure S13	^{13}C NMR spectrum of compound **3**	15
Figure S14	ESI-MS spectrum of compound **3**	16
Figure S15	^{1}H NMR spectrum of compound **4**	17
Figure S16	^{13}C NMR spectrum of compound **4**	18
Figure S17	ESI-MS spectrum of compound **4**	19
Figure S18	^{1}H NMR spectrum of compound **5**	20
Figure S19	^{13}C NMR spectrum of compound **5**	21
Figure S20	ESI-MS spectrum of compound **5**	22
Figure S21	^{1}H NMR spectrum of compound **6**	23
Figure S22	^{13}C NMR spectrum of compound **6**	24
Figure S23	ESI-MS spectrum of compound **6**	25
Figure S24	^{1}H NMR spectrum of compound **7**	26
Figure S25	^{13}C NMR spectrum of compound **7**	27
Figure S26	ESI-MS spectrum of compound **7**	28

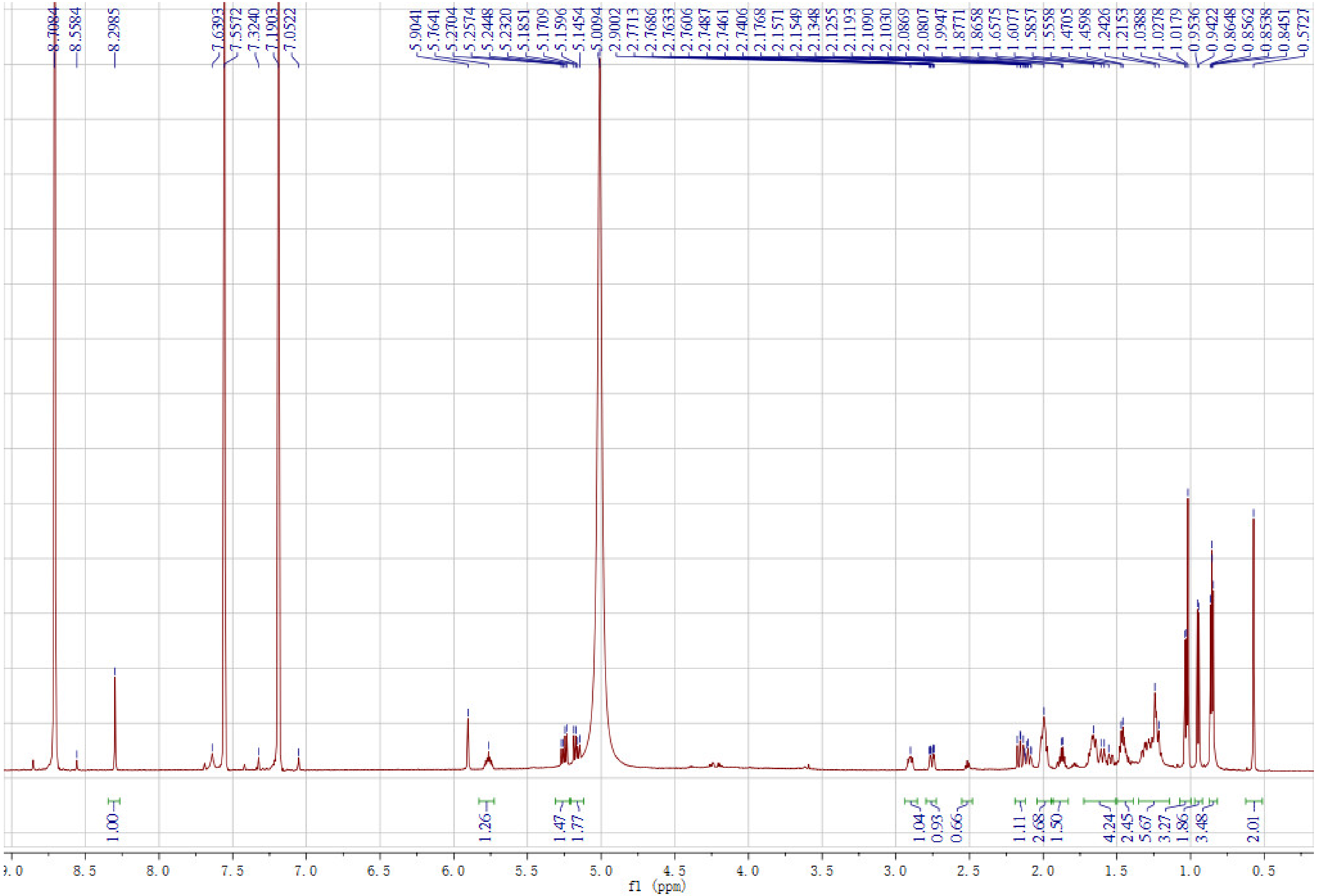

Figure S1. ^{1}H NMR spectrum of compound **1**

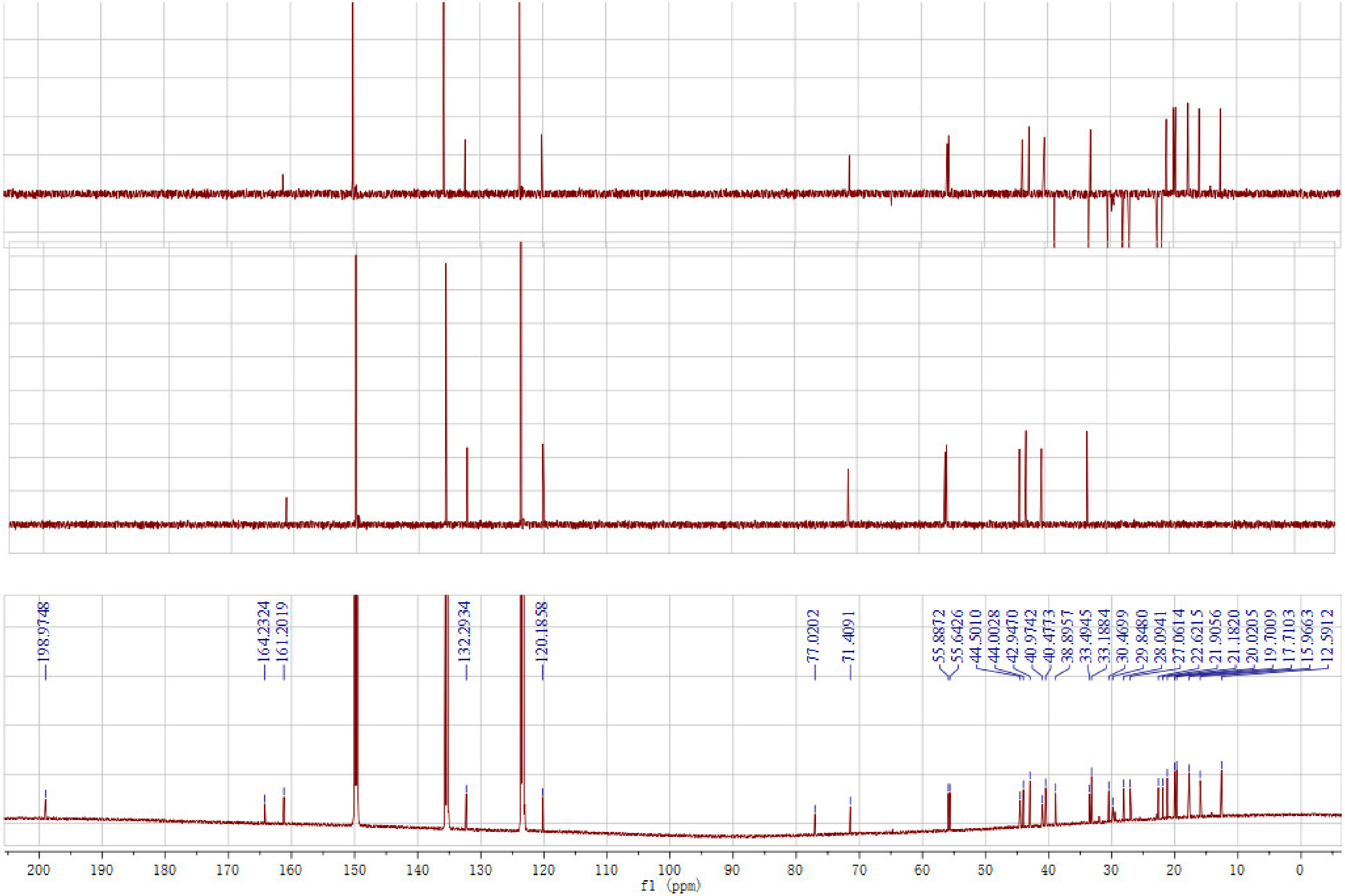

Figure S2. ^{13}C NMR spectrum of compound **1**

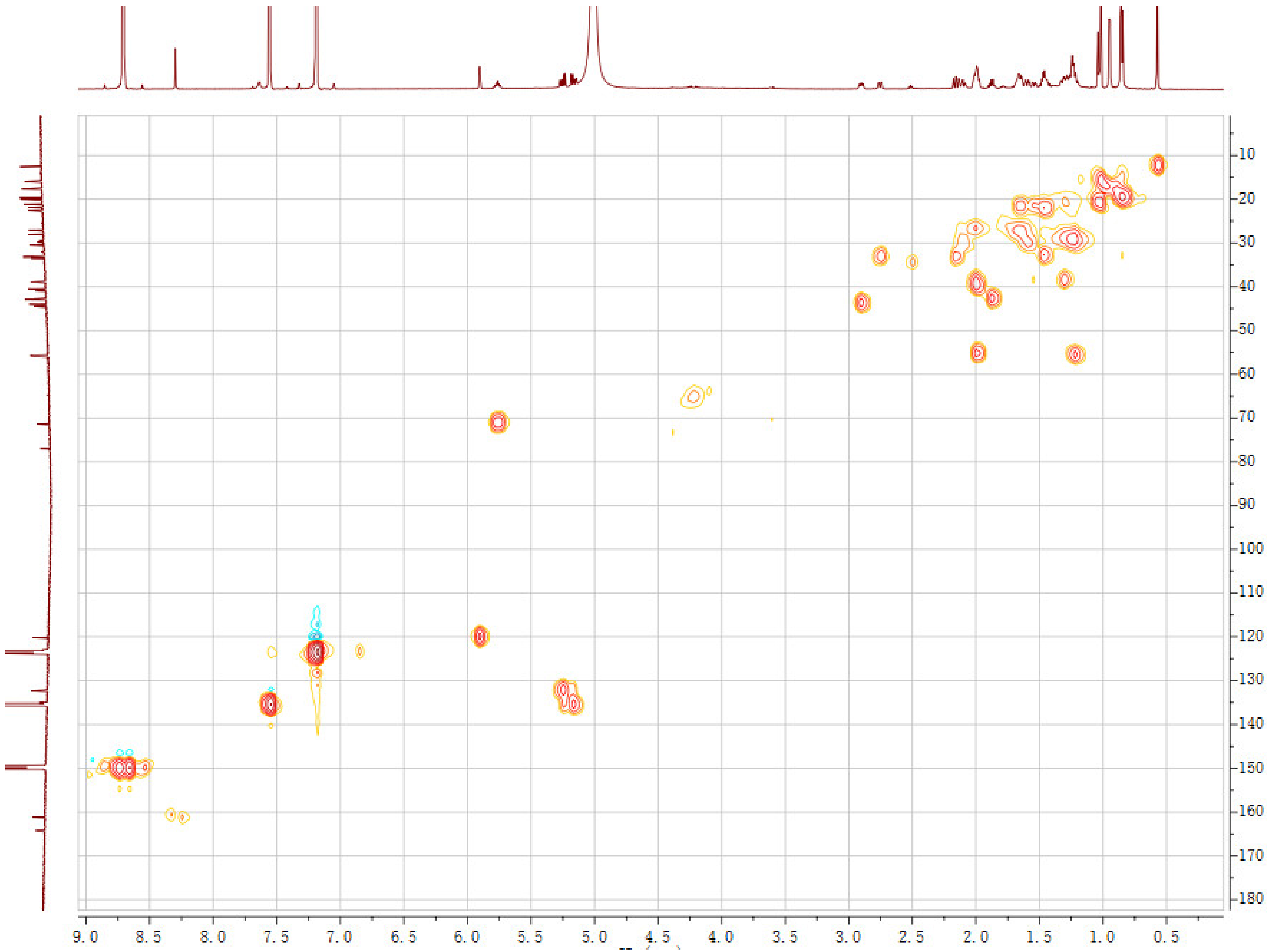

Figure S3. HSQC spectrum of compound **1**

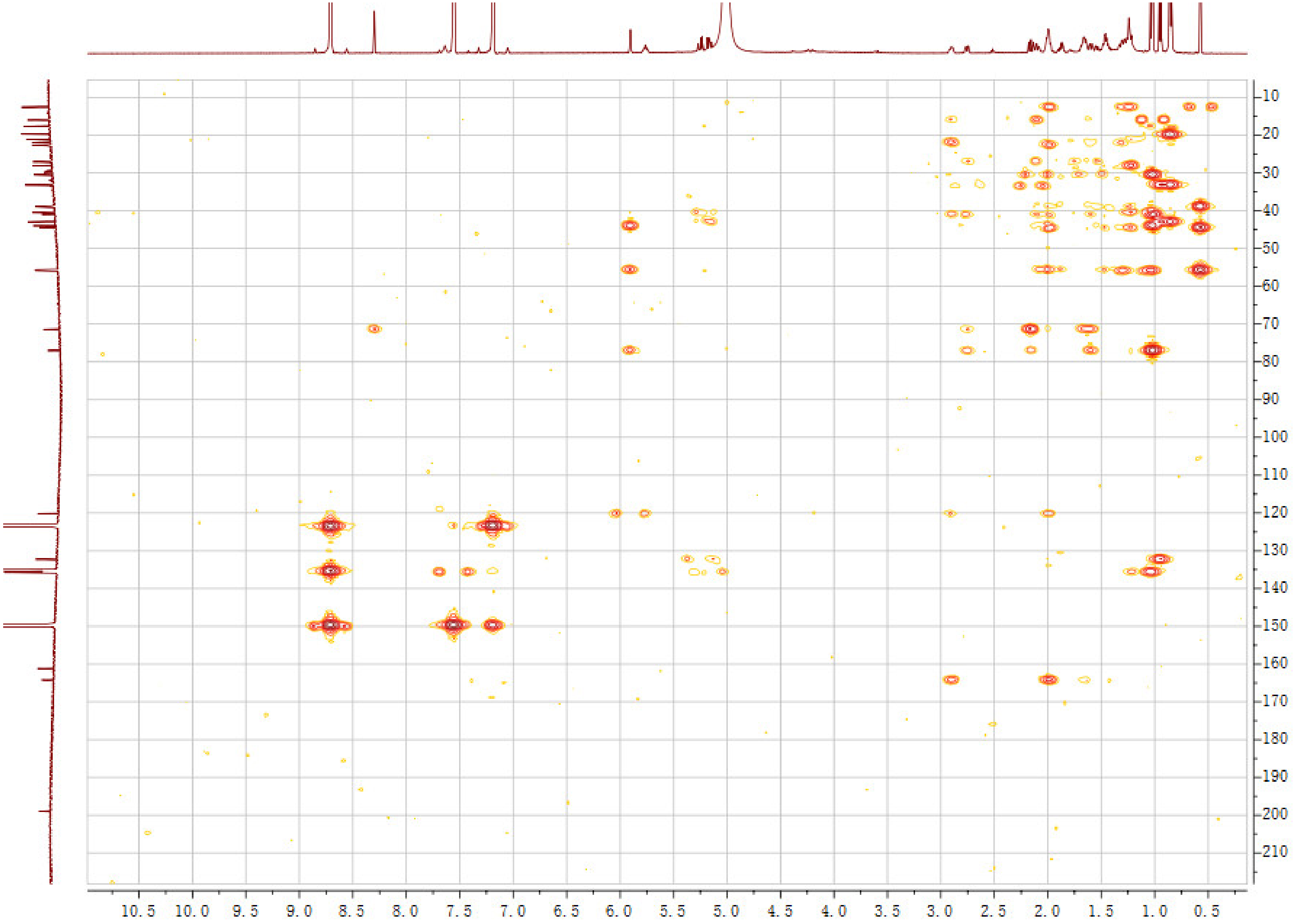

Figure S4. HMBC spectrum of compound **1**

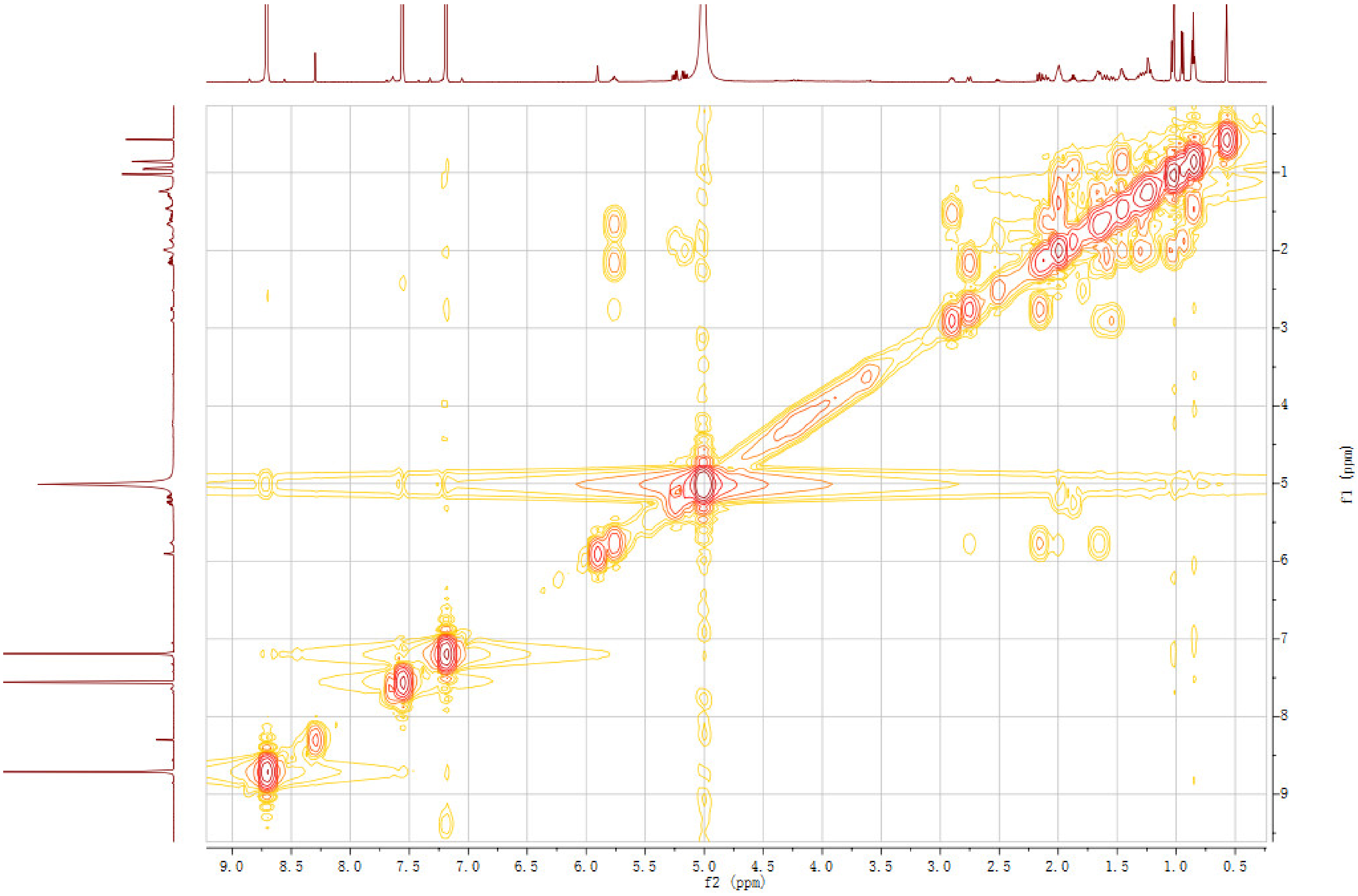

Figure S5. ^{1}H–^{1}H COSY spectrum of compound **1**

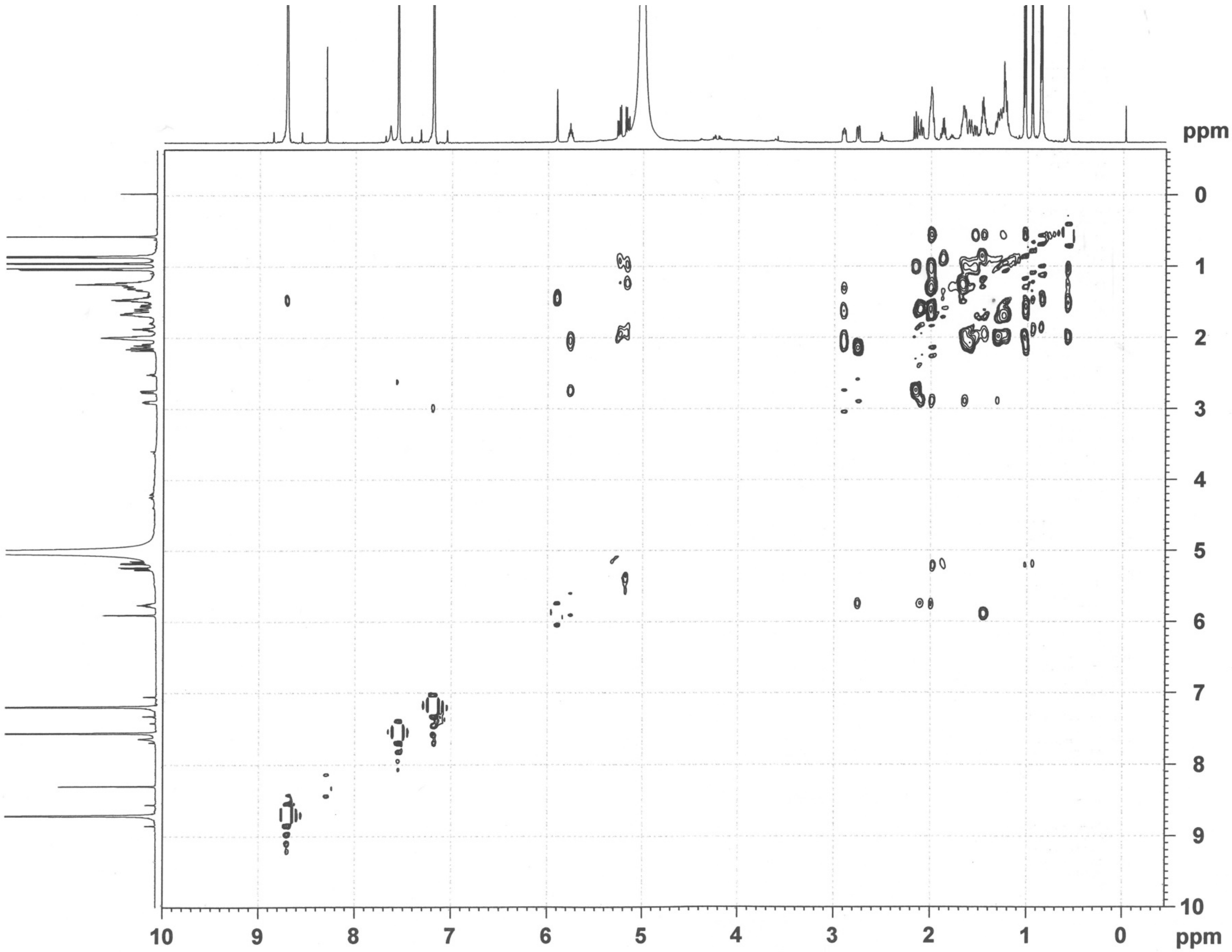

Figure S6. ROESY spectrum of compound **1**

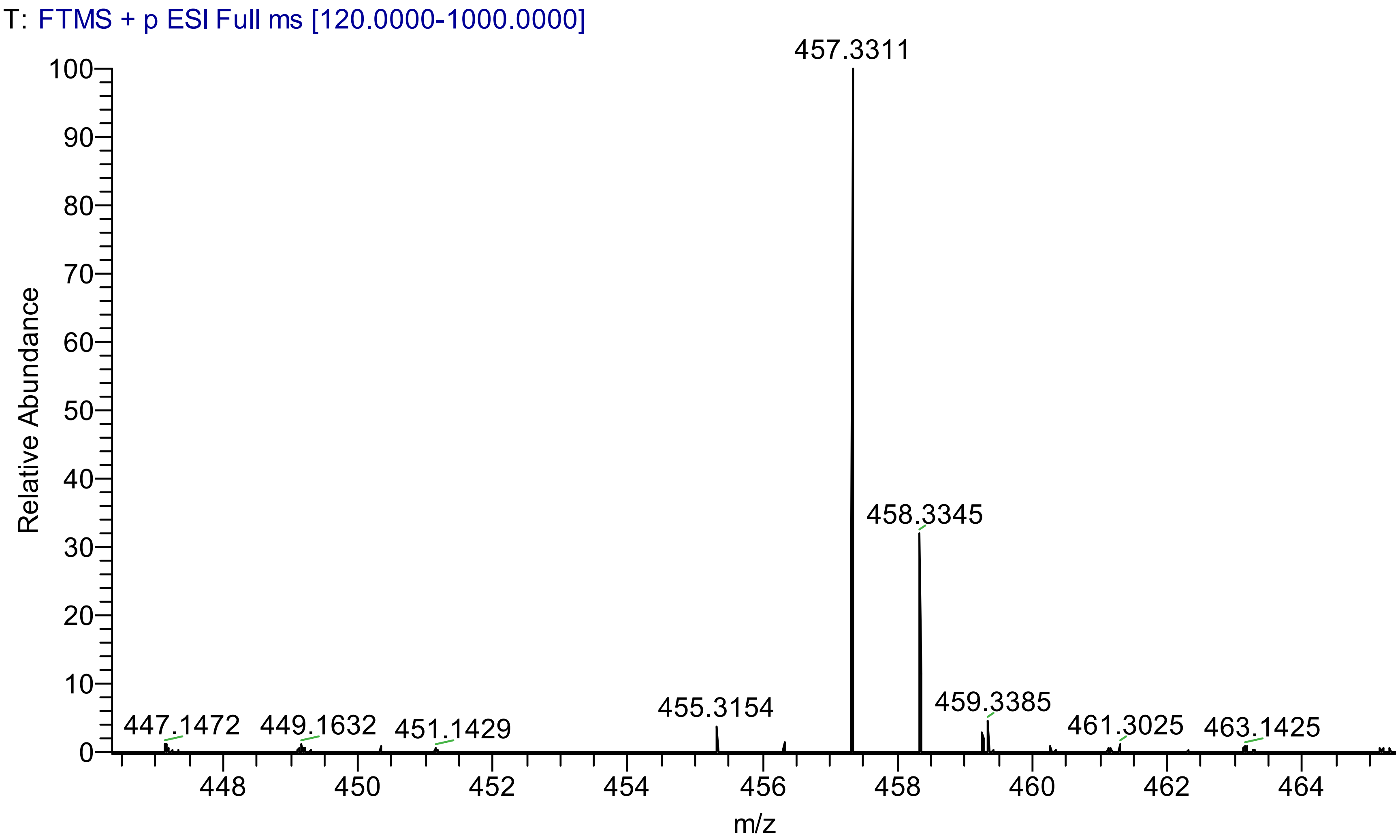

m/z	Theo. Mass	Delta (mmu)	RDB equiv.	Composition
**457.3311**	**457.3312**	**-0.14**	**7.5**	**$C_{29}H_{45}O_{4}$**

Figure S7. HR-ESI-MS of compound **1**

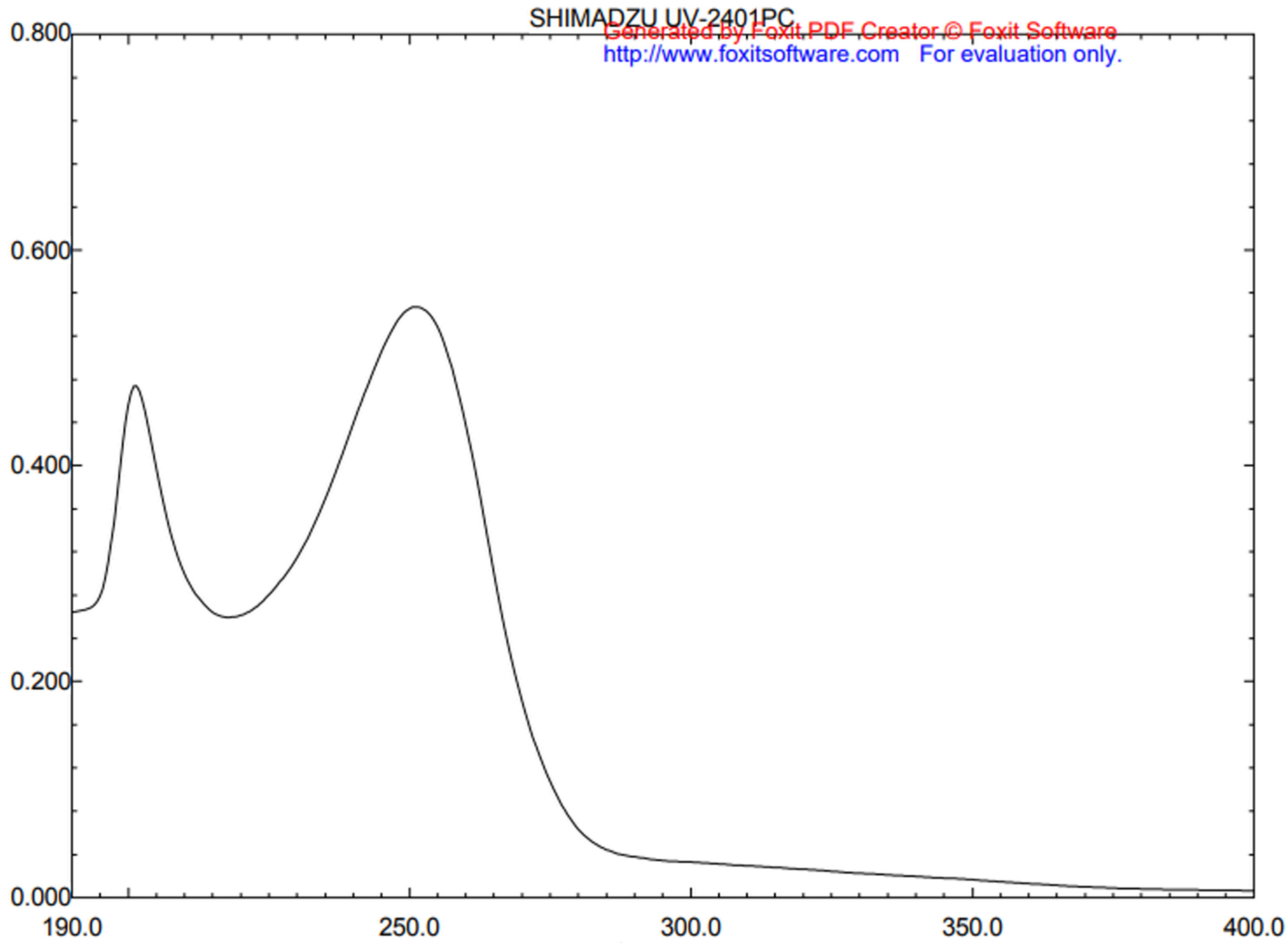

Generated by Foxit PDF Creator © Foxit Software
http://www.foxitsoftware.com For evaluation only.

Wavelength (nm.)

Figure S8.UV spectrum of compound **1**

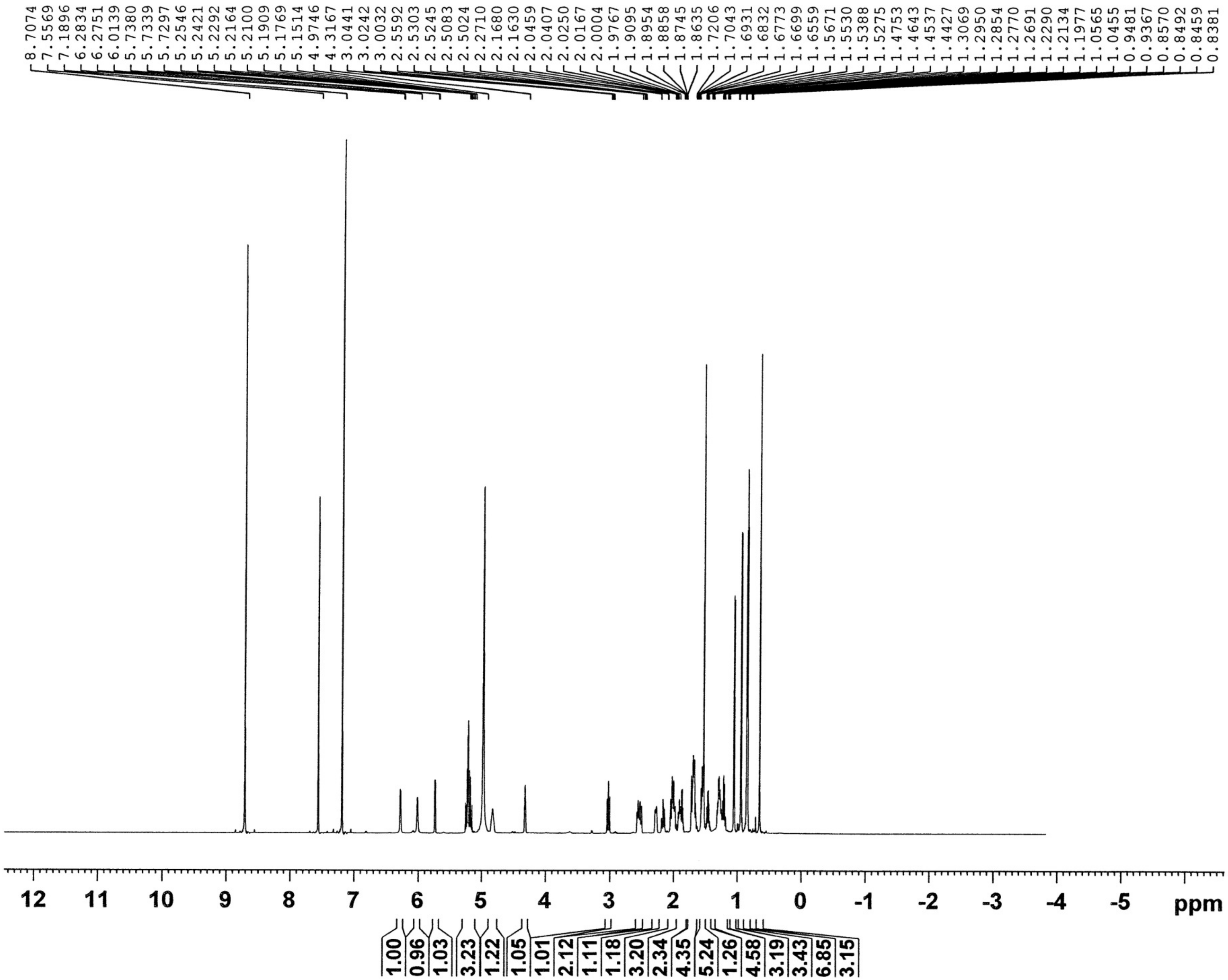

Figure S9. ^{1}H NMR spectrum of compound **2**

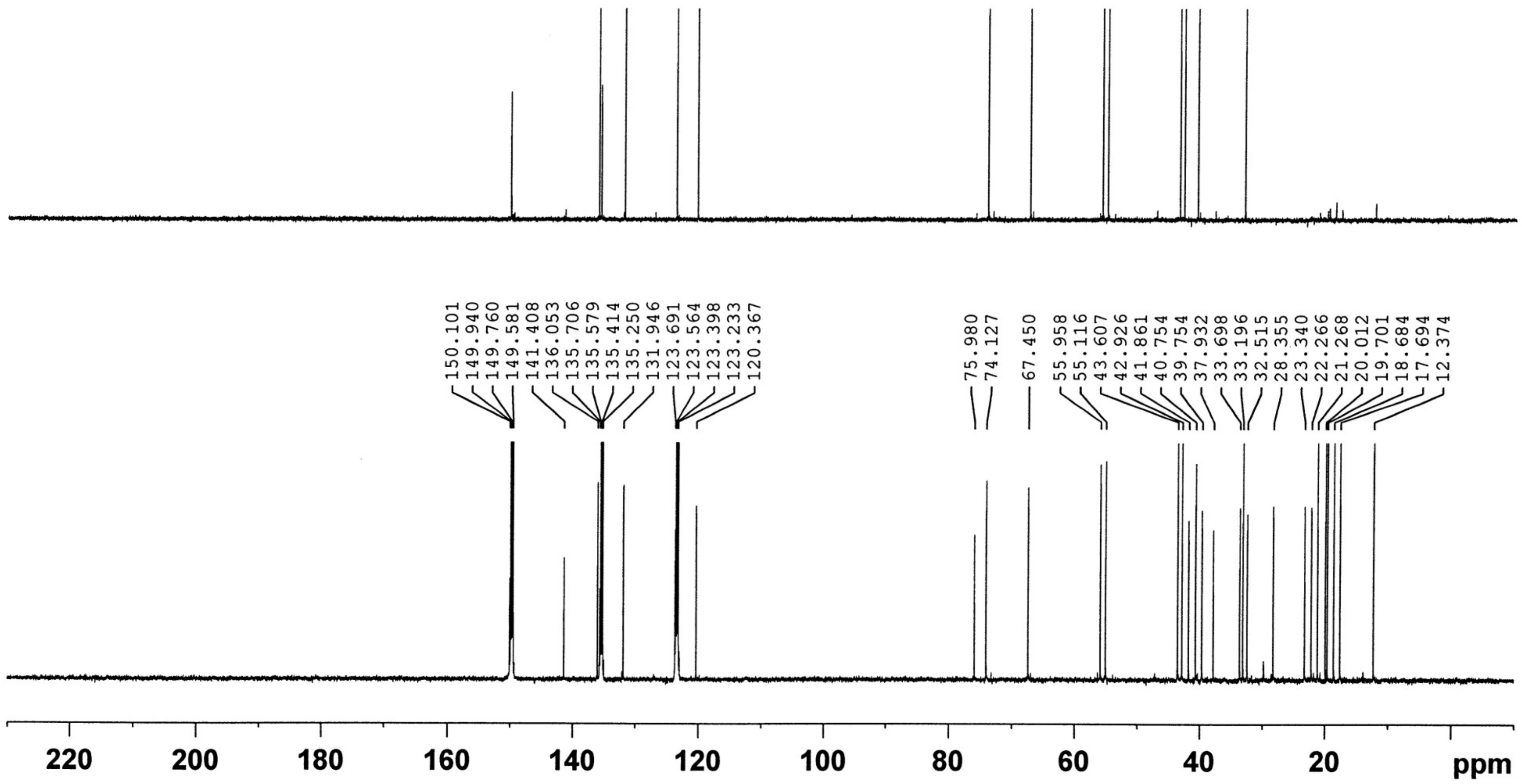

Figure S10. ^{13}C NMR spectrum of compound **2**

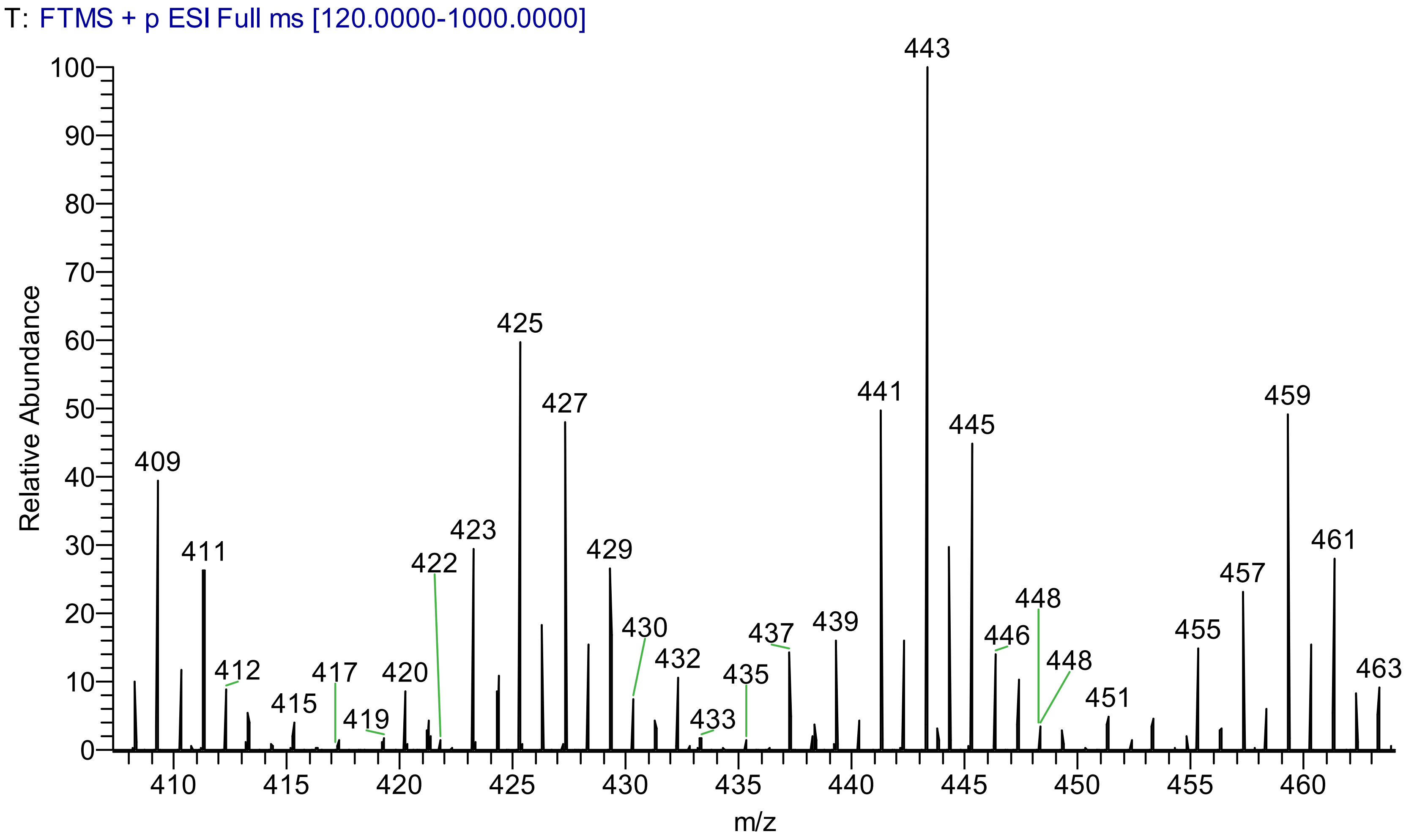

Figure S11. ESI-MS spectrum of compound **2**

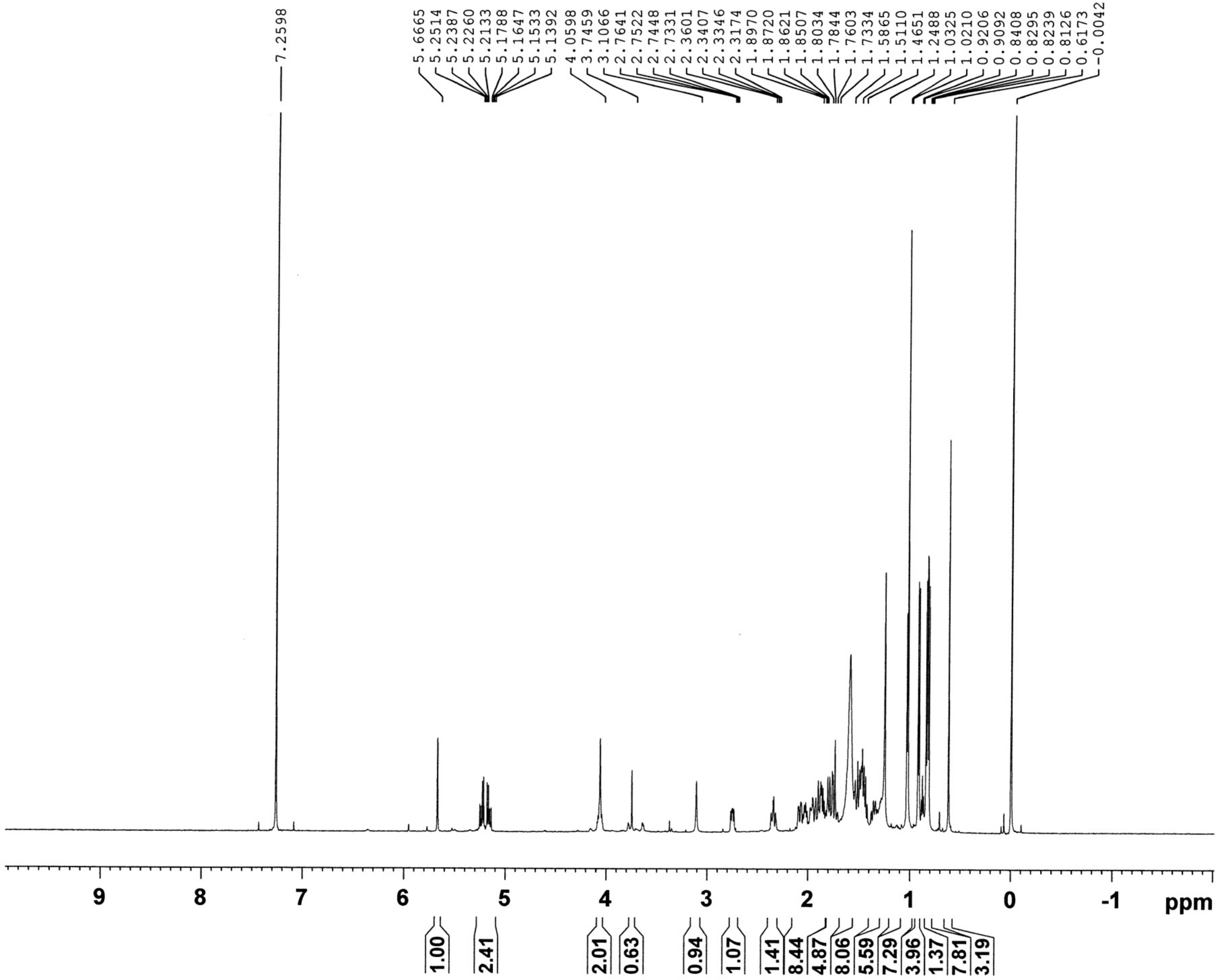

Figure S12. ^{1}H NMR spectrum of compound **3**

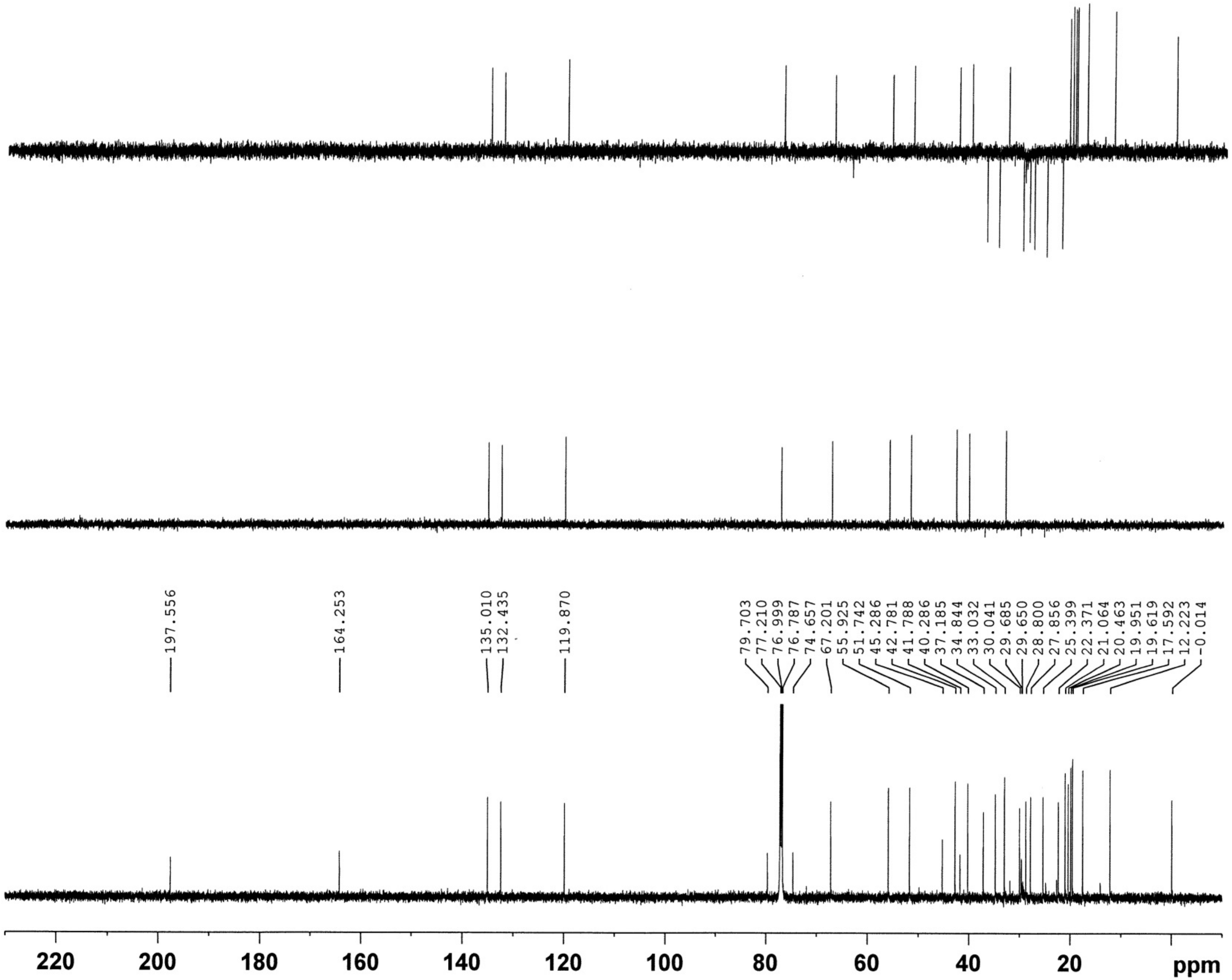

Figure S13. ${}^{13}C$ NMR spectrum of compound **3**

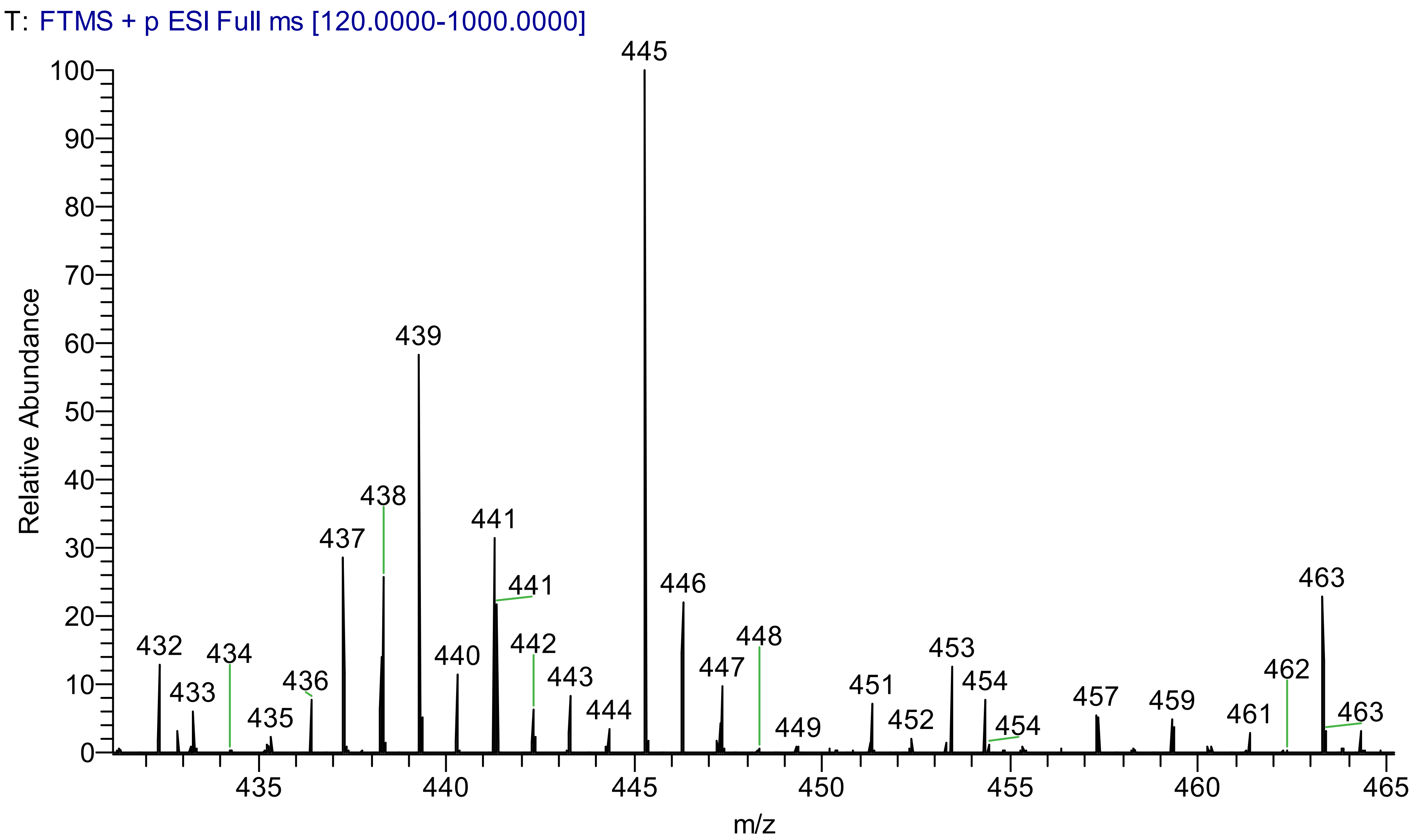

Figure S14. ESI-MS spectrum of compound **3**

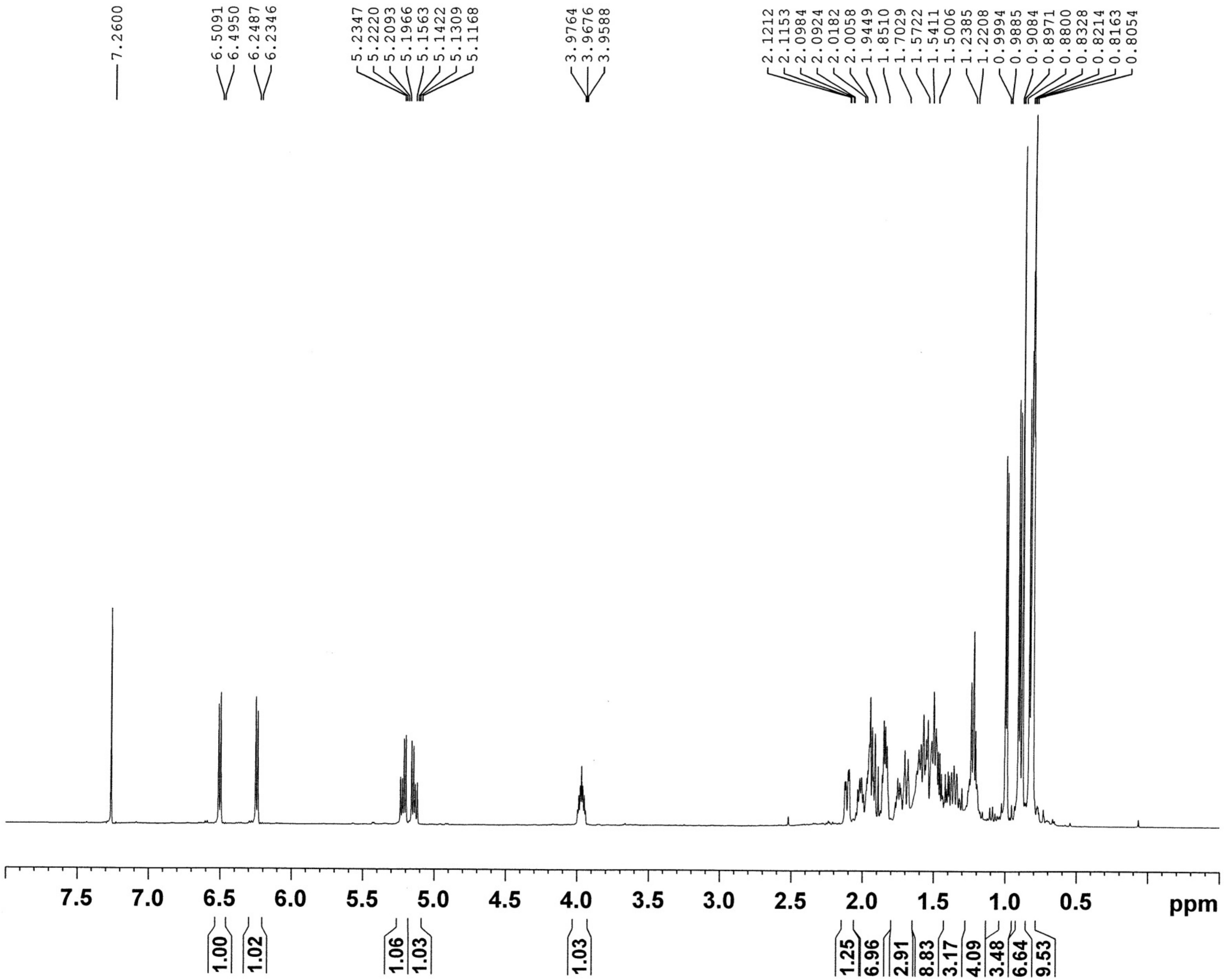

Figure S15. ^{1}H NMR spectrum of compound **4**

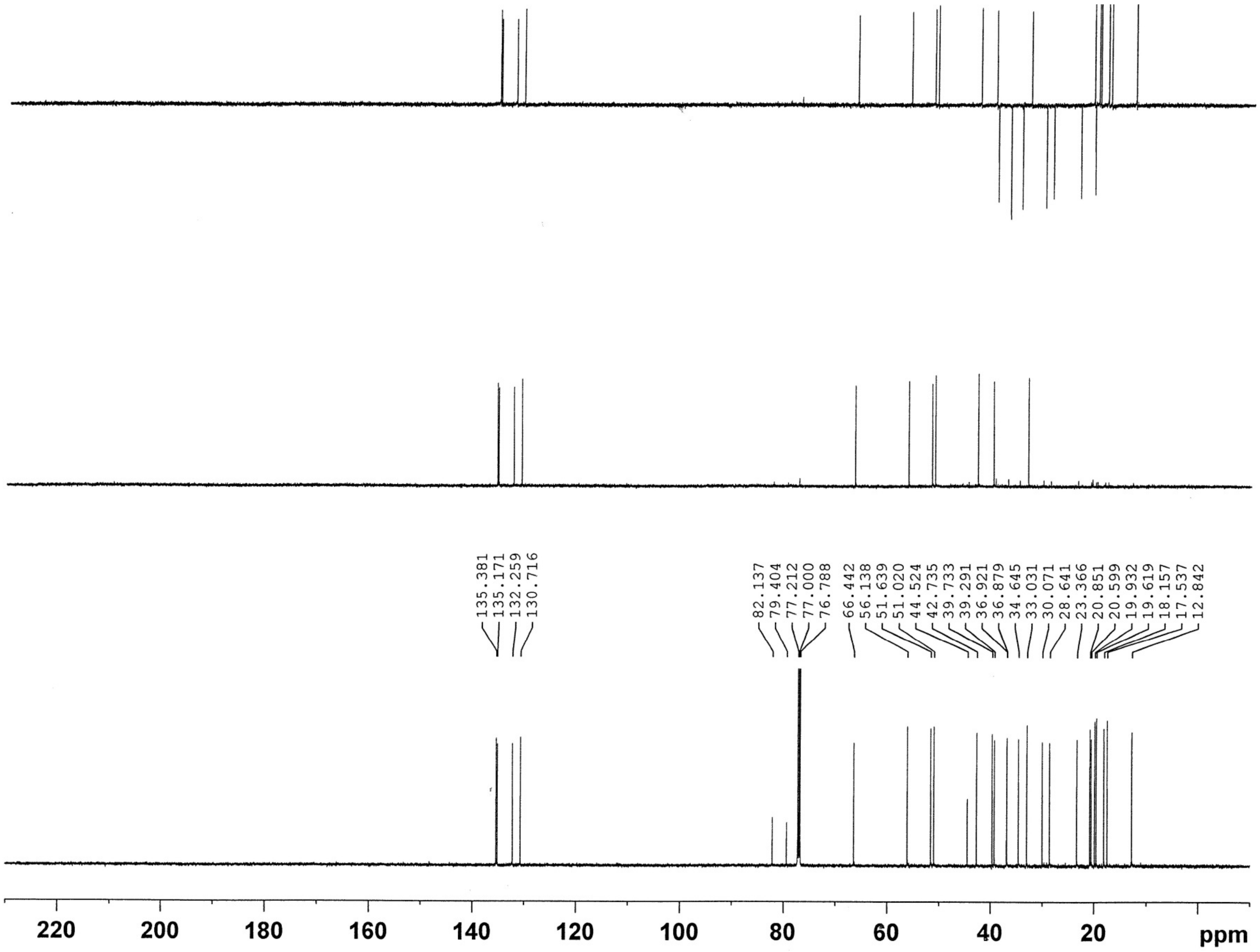

Figure S16. ^{13}C NMR spectrum of compound **4**

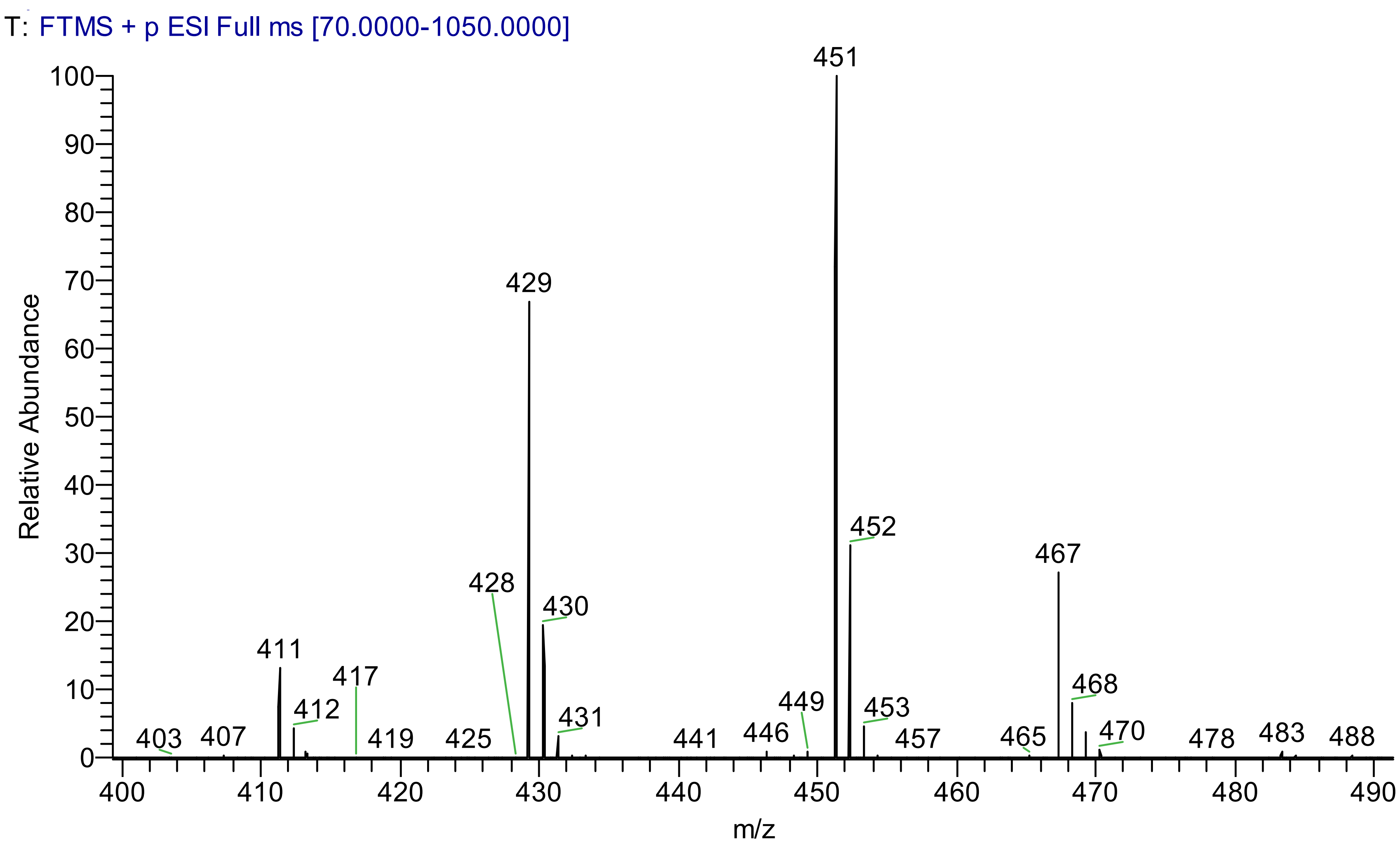

Figure S17. ESI-MS spectrum of compound **4**

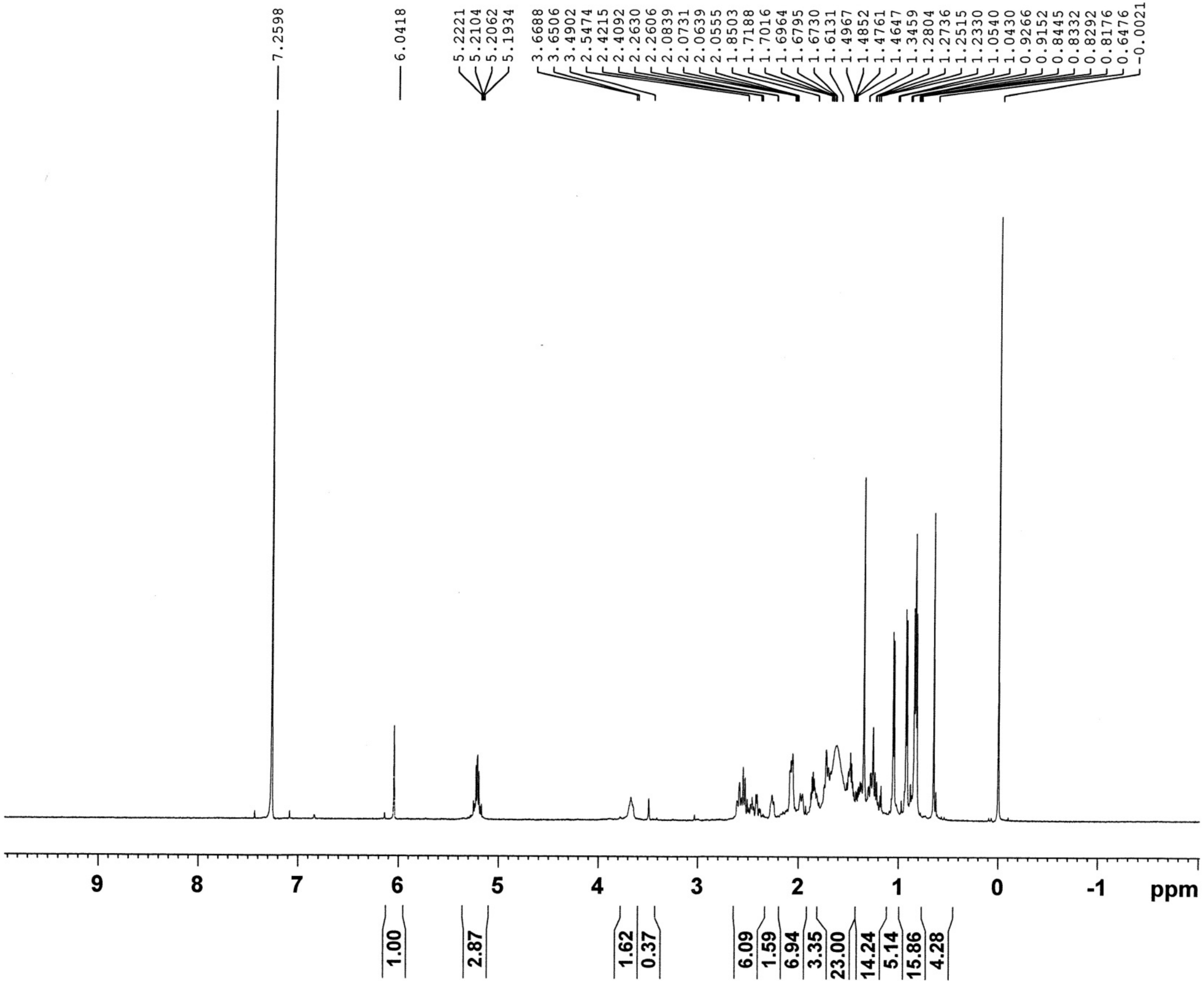

Figure S18. ^{1}H NMR spectrum of compound **5**

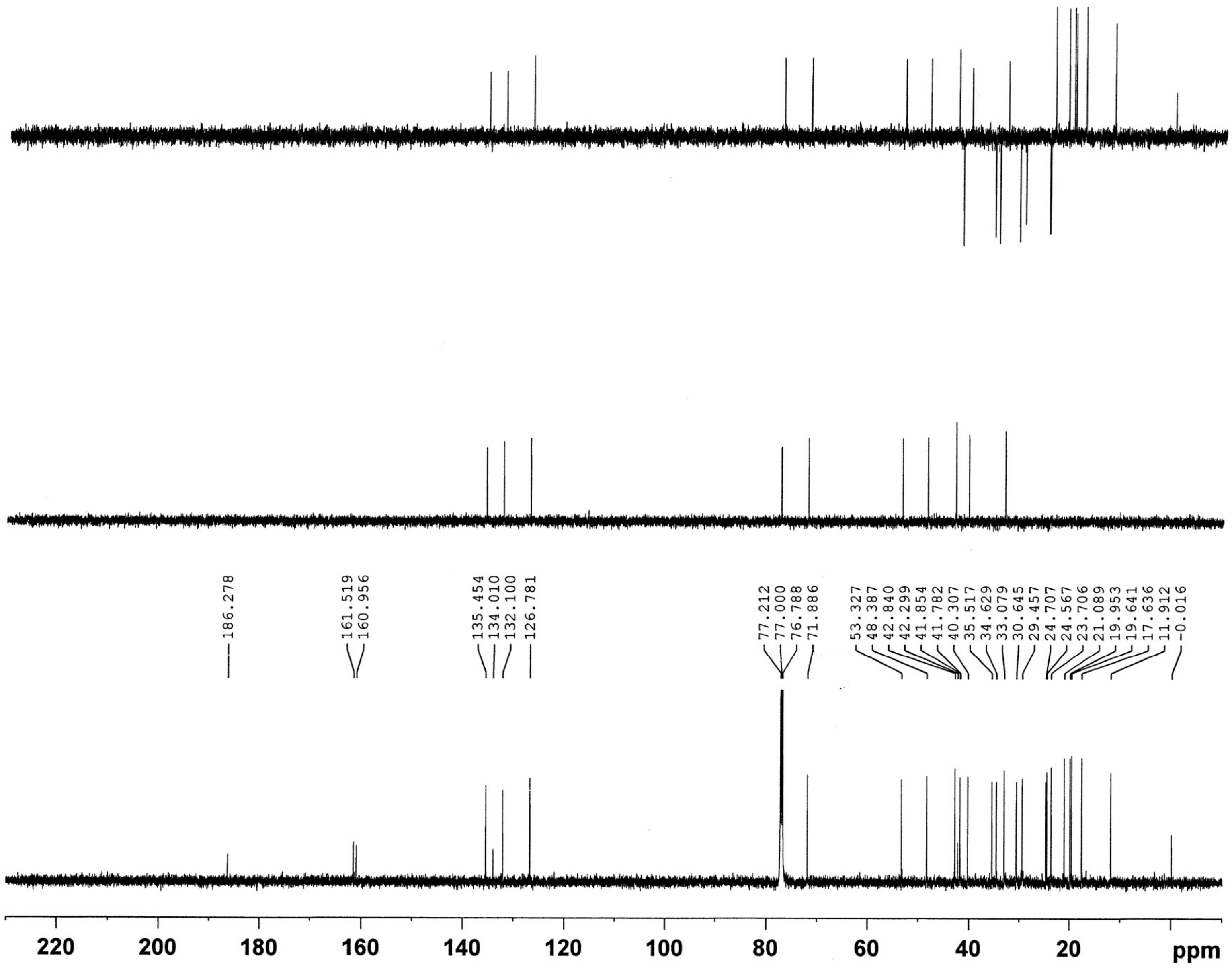

Figure S19. ^{13}C NMR spectrum of compound **5**

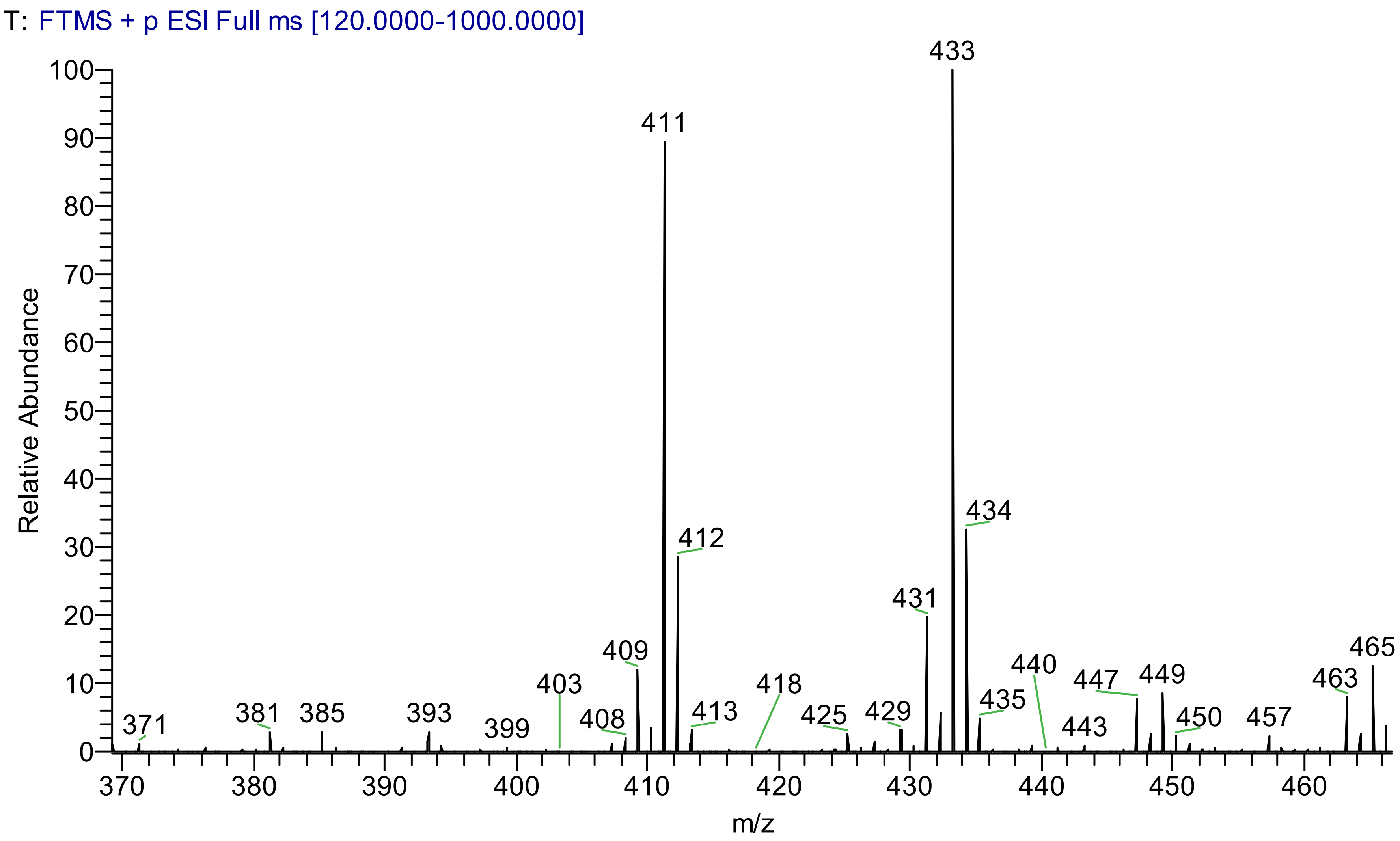

Figure S20. ESI-MS spectrum of compound **5**

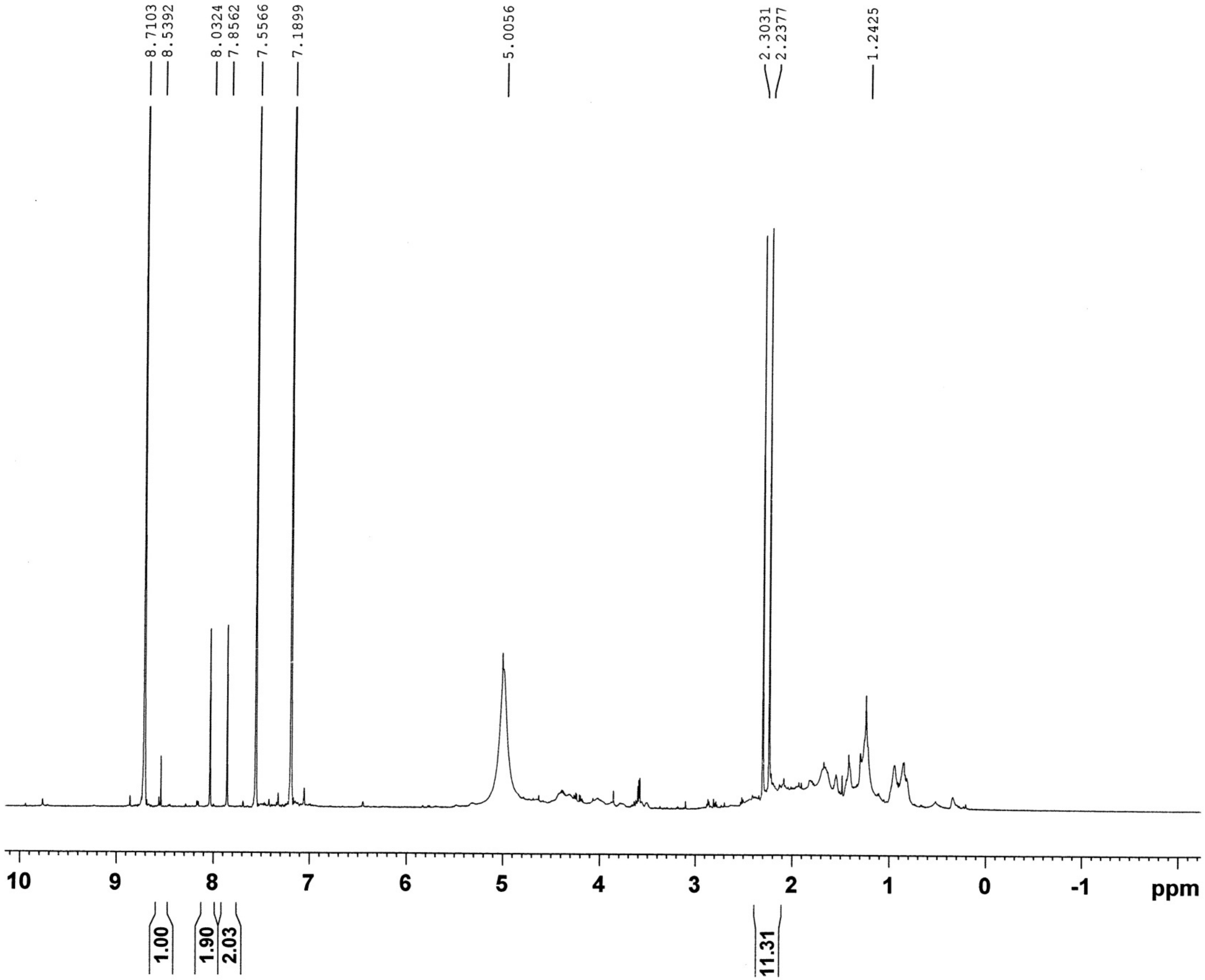

Figure S21. ^{1}H NMR spectrum of compound **6**

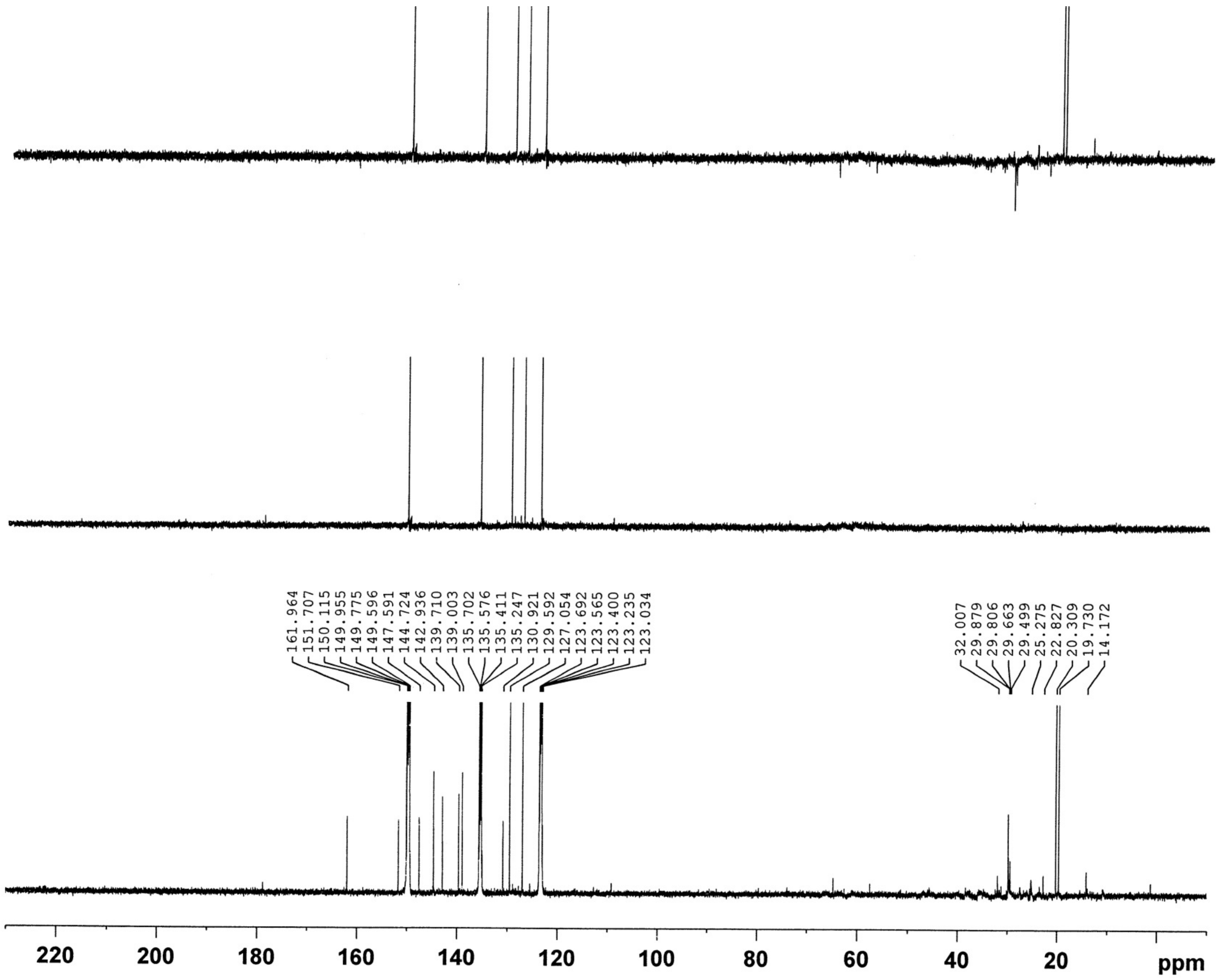

Figure S22. ^{13}C NMR spectrum of compound **6**

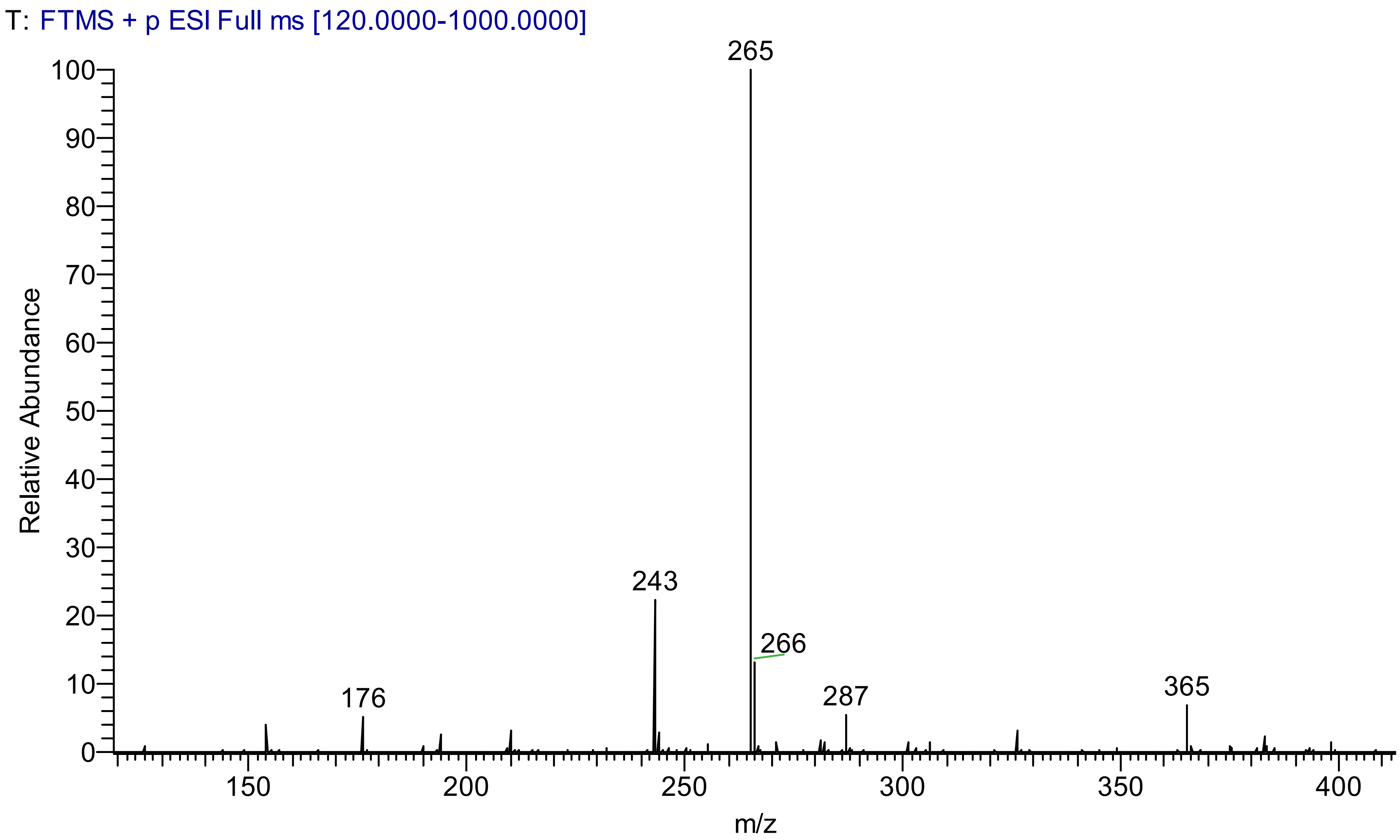

Figure S23. ESI-MS spectrum of compound **6**

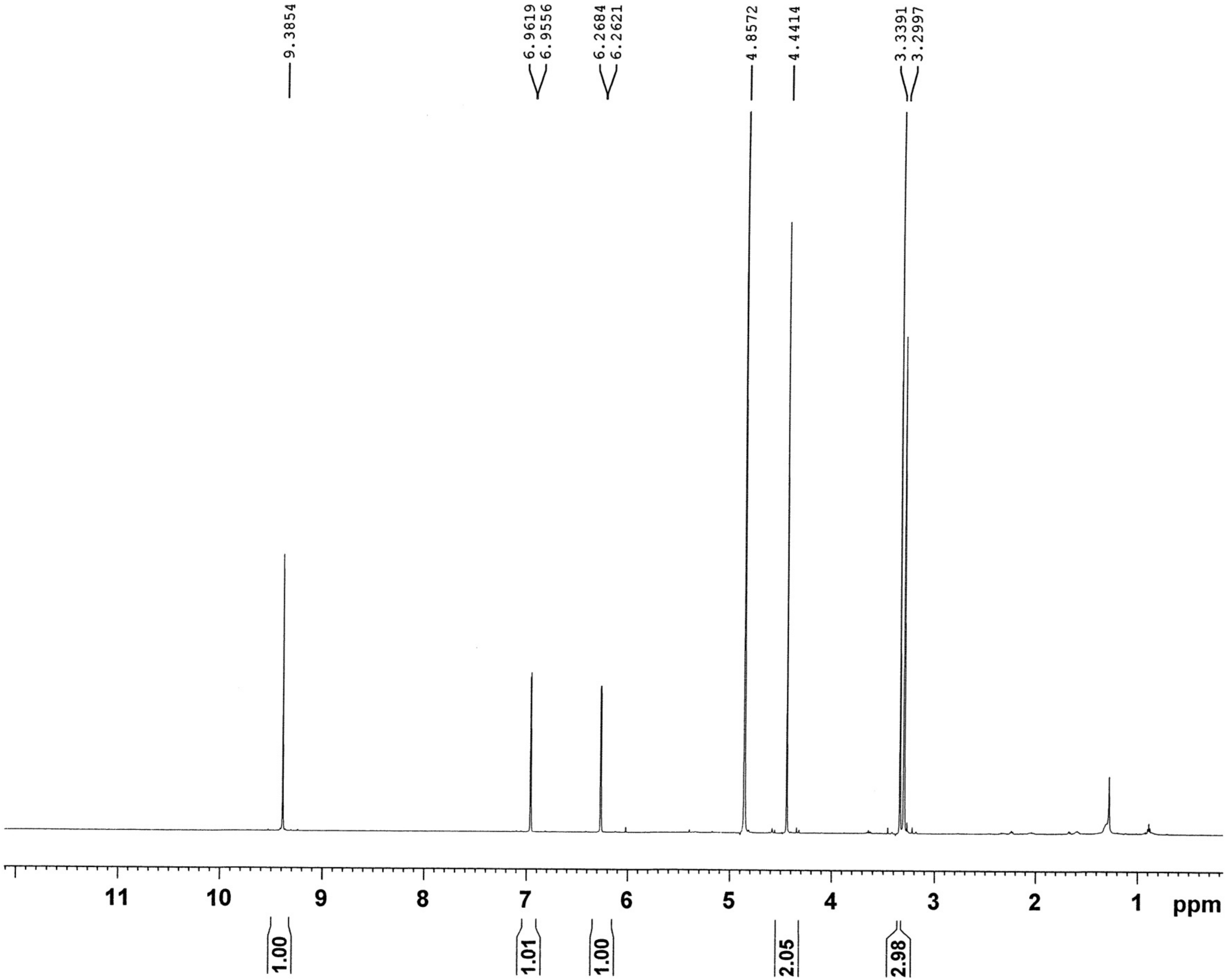

Figure S24. ^{1}H NMR spectrum of compound **7**

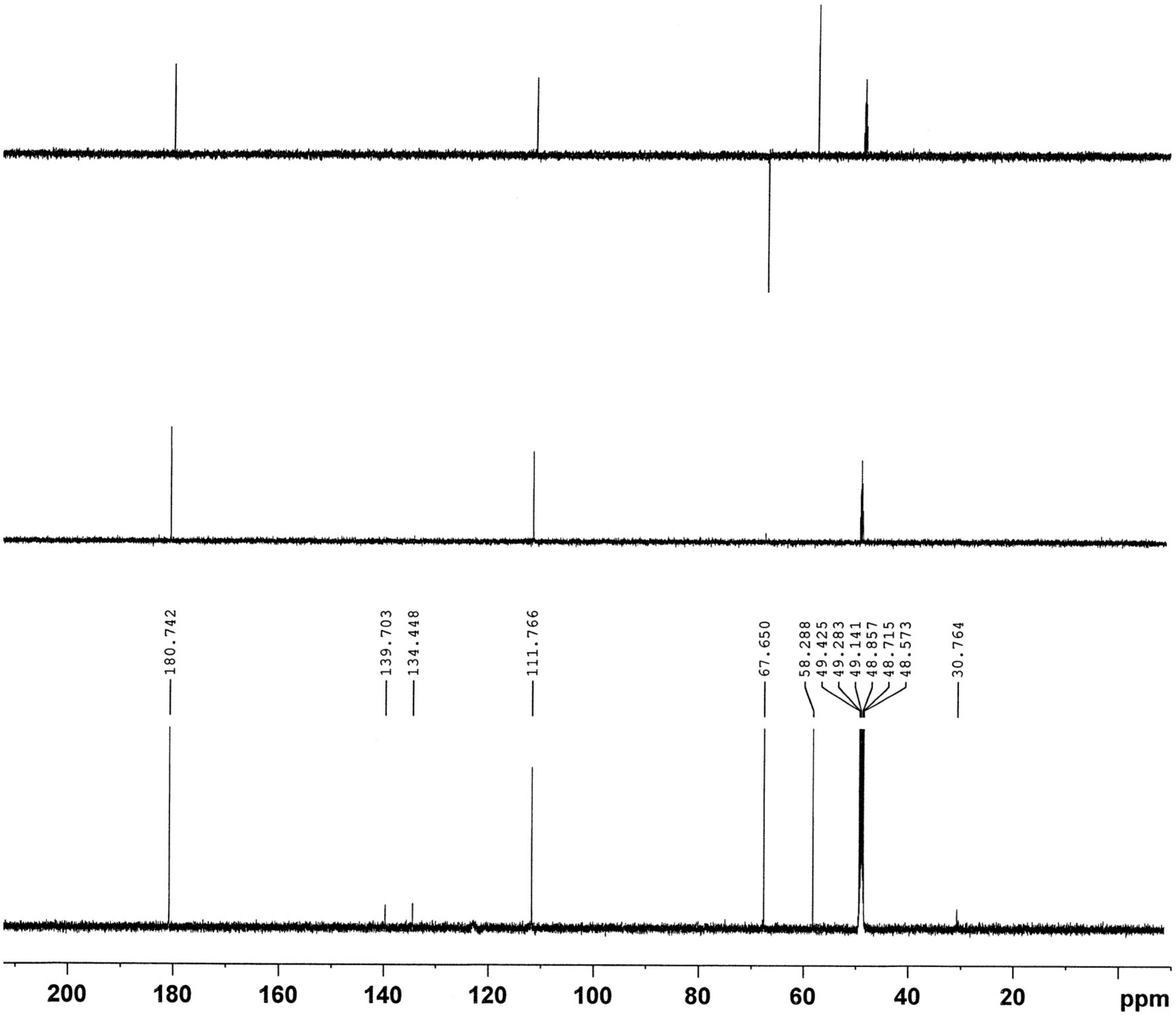

Figure S25. ^{13}C NMR spectrum of compound **7**

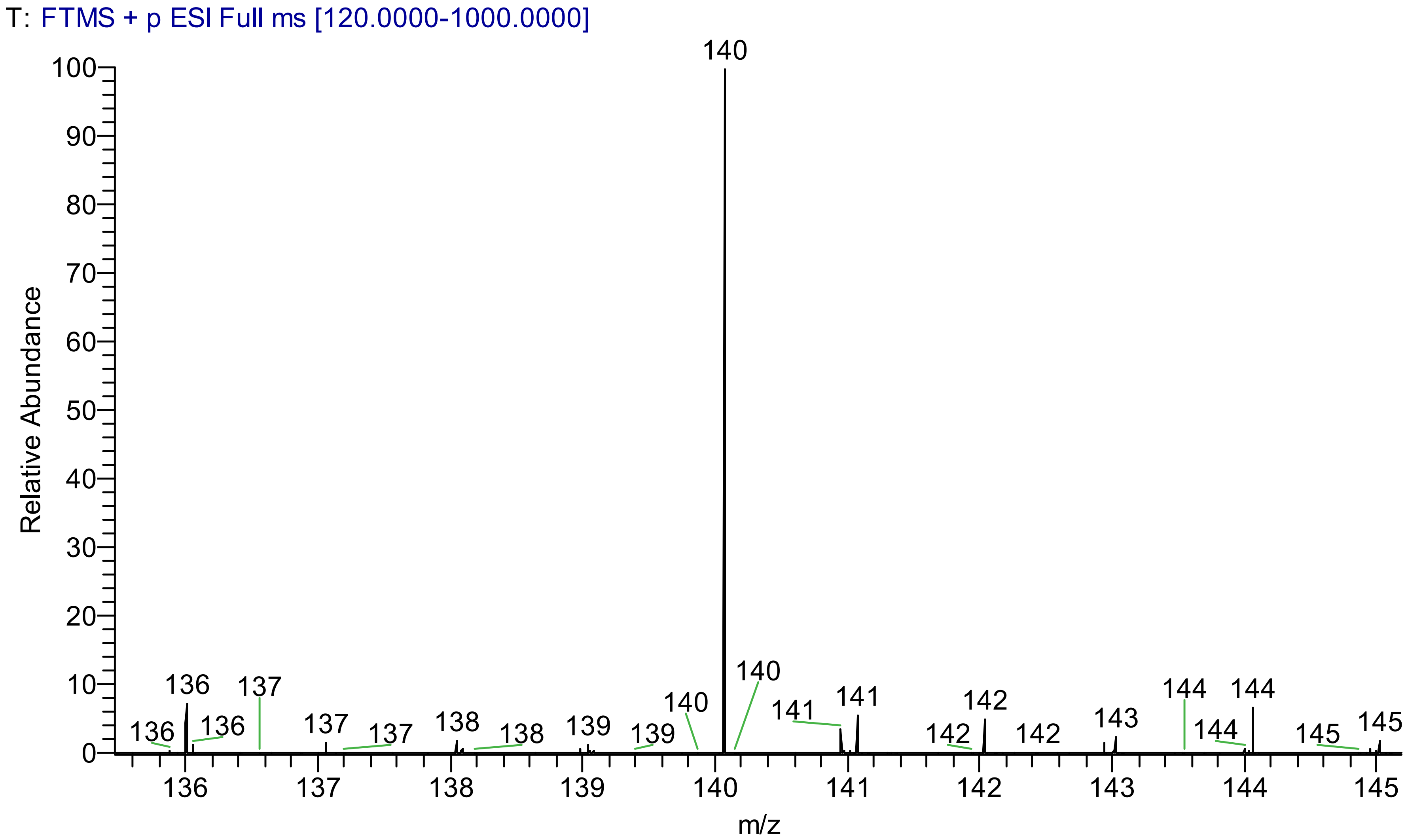

Figure S26. ESI-MS spectrum of compound **7.**
